# Pathogen spectrum and immunotherapy in patients with anti-IFN-γ autoantibodies: A multicenter retrospective study and systematic review

**DOI:** 10.3389/fimmu.2022.1051673

**Published:** 2022-12-08

**Authors:** Ye Qiu, Gaoneng Fang, Feng Ye, Wen Zeng, Mengxin Tang, Xuan Wei, Jinglu Yang, Zhengtu Li, Jianquan Zhang

**Affiliations:** ^1^ State Key Laboratory of Respiratory Disease, National Clinical Research Center for Respiratory Disease, Guangzhou Institute of Respiratory Health, the First Affiliated Hospital of Guangzhou Medical University, Guangzhou, China; ^2^ Department of Respiratory and Critical Medicine, The Eighth Affiliated Hospital, Sun Yat-Sen University, Shenzhen, Guangdong, China; ^3^ Department of Respiratory and Critical Medicine, The First Affiliated Hospital of Guangxi Medical University, Nanning, Guangxi, China; ^4^ Department of General medicine, The Cancer Affiliated Hospital of Guangxi Medical University, Nanning, Guangxi, China

**Keywords:** anti-IFN-γ autoantibody, epidemiology, immunodeficiency, immunotherapy pathogen spectrum, immunotherapy

## Abstract

**Background:**

Anti-interferon-γ autoantibody (AIGA) positivity is an emerging immunodeficiency syndrome closely associated with intracellular infection in individuals without human immunodeficiency virus (HIV). However, the information on epidemiology, pathogen spectrum, and immunotherapy among these patients lack a systematic description of large data.

**Methods:**

This systematic literature review and multicenter retrospective study aimed to describe the pathogen spectrum and review treatment strategies among patients with AIGA positivity.

**Results:**

We included 810 HIV-negative patients with AIGA positivity infected with one or more intracellular pathogens. Excluding four teenagers, all the patients were adults. The most common pathogen was nontuberculous mycobacteria (NTM) (676/810, 83.5%). A total of 765 NTM isolates were identified in 676 patients with NTM, including 342 (44.7%) rapid-grower mycobacteria, 273 (35.7%) slow-grower mycobacteria, and 150 (19.6%) unidentified NTM subtype. Even with long-term and intensive antimicrobial treatments, 42.6% of patients with AIGA positivity had recurrence and/or persistent infection. Sixty-seven patients underwent immunoregulatory or immunosuppressive therapy, and most (60) achieved remission. The most common treatment strategy was rituximab (27/67, 40.3%) and cyclophosphamide (22/67, 32.8%), followed by cyclophosphamide combined with glucocorticoids (8/67, 11.9%).

**Conclusions:**

Intracellular pathogen was the most common infection in patients with AIGA positivity. The predominant infection phenotypes were NTM, varicella-zoster virus, *Talaromyces marneffei*, and *Salmonella* spp., with or without other opportunistic infections. AIGA immunotherapy, including rituximab or cyclophosphamide, has yielded good preliminary results in some cases.

## Introduction

Since the first case of anti-interferon-γ autoantibody (AIGA) positive patient was reported in 2004, AIGA-related adult-onset immunodeficiency, considered as an autoimmune phenocopy of inborn genetic errors of the IL-12/IFN-γ axis (1), has closely been associated with nontuberculous mycobacteria (NTM) and *varicella zoster virus* in individuals without human immunodeficiency virus (HIV) in Southeast Asia (2). However, a new strong relationship was observed between AIGA positivity and other intracellular pathogens, such as *Talaromyces marneffei* and *Salmonella* spp (2, 3)., *Cryptococcus* spp., *Mycobacterium tuberculosis*, *Burkholderia* spp., *Epstein–Barr virus*, and *Histoplasma capsulatum*, has been reported (3, 4). In addition, AIGA-positive patients are not exclusive to Southeast Asia. AIGA-positivity has also been reported in America, the United Kingdom, and Germany (5–7).

Recently, AIGA positivity has been regarded as an important susceptibility factor and prognostic indicator for intracellular infection in patients without HIV (2, 3, 8). These patients were more likely to have a poor prognosis and heightened inflammatory response (9). The immune deficiency mechanism of this anti-cytokine autoantibody may be related to the neutralizing effect on IFN-γ inhibit STAT1 phosphorylation and IL-12 production (8–10), damaging the antimicrobial immunity in macrophages by inhibiting M1 macrophage polarization and blocking the production of proinflammatory factors, including cytokines/chemokines and reactive oxygen/nitrogen species (11). Therefore, effectively reducing the titer of AIGA may improve infection prognosis and reduce recurrence and double or multiple infections.

An increasing number of reports have focused on AIGA-related adult-onset immunodeficiency versatile clinical presentations, diagnosis, and treatment. However, to our knowledge, understanding of the epidemiology, pathogen spectrum, diagnostic criteria, and immunotherapy (including immunoregulatory or immunosuppressive therapy) of AIGA is limited, and there is a lack of a systematic description of this disease based on large data. Therefore, this study aimed to describe the epidemiological characteristics and pathogen spectrum of AIGA positivity and review the immunotherapeutic strategies developed for these patients to provide deeper insight into AIGA positivity and guidance for physicians in clinical practice.

## Methods

### Multicenter retrospective study in guangxi and guangdong

We conducted a four-year (January 2018 to September 2021) multicenter retrospective study in Guangxi and Guangdong, China, at 14 participating centers. Complete histories were obtained, including demographic data, geographical distribution, infecting pathogens, and prognoses. The Ethics Review Board of the First Affiliated Hospital of Guangxi Medical University approved the study (2018.KY-E-094), which was conducted in accordance with Good Clinical Practice and the Declaration of Helsinki.

The inclusion criteria for patients in retrospective studies in Guangxi and Guangdong were as follows: 1) positive AIGA; 2) confirmed pathogen infection *via* microbiological culture, pathological examination, metagenomics next-generation sequencing and/or polymerase chain reaction; and 3) patients who provided informed consent for participation. We conducted a retrospective review of the medical records of patients with AIGA positivity. Healthy volunteers and patients with tuberculosis were recruited from the physical examination center at the same hospital.

### AIGA assay

Serum samples were obtained under sterile conditions and stored in a serum bank at -80°C. The AIGA in the serum was measured by an enzyme-linked immunosorbent assay (ELISA) kit (Cloud-Clone Corp., Wuhan, China), with a detection range of 12–200 ng/mL. Then, we tested the optical density (OD) of gradient dilution concentrations of standards. Finally, the standard fitting curve was applied based on the classic software of Curve Expert 1.4. After confirming that the kit test was working normally, we conducted a preliminary experiment and found the most suitable dilution concentration to detect the sample. The serum samples from patients and a healthy control were diluted at 1:1500 and 1:600, respectively, with phosphate-buffered saline. Throughout the process, we strictly adhered to the manufacturer’s protocol regarding the preparation and testing of the standards and samples. The concentration of AIGA in the sample is determined by comparing the OD of the sample to the standard curve (2, 4, 9, 12).

### Definition of patients with AIGA positivity

All 79 patients, including 66 from Guangxi and 13 from Guangdong, were assessed for neutralization activity. AIGA disease requires (1) the detection of AIGA by ELISA and (2) an assessment of the neutralizing activity of AIGA by assays of STAT-1 phosphorylation or human leukocyte antigen (HLA)-DR expression on IFN-γ-responsive cells by flow cytometry (3, 10).

The normal range for the AIGA concentration was estimated using the log-normal distribution, defined by the 99th percentile from the 103 healthy volunteers and 30 patients with tuberculosis. Outlying concentrations were classified as positive for AIGA (2, 8). The mean titer of AIGA among these 133 participants was 3033.81 ± 1576.31ng/mL, and the 99th percentile concentration was 6502.53 ng/mL. Therefore, it is positive when the AIGA titer exceeds 6502.53 ng/mL.

### Systematic literature review search strategy

We searched PubMed, Web of Science, Embase, and BIOSIS Library for original case reports and cohort studies on AIGA published in English between January 1, 2004 and September 31, 2021. We used the keywords “anti-interferon (IFN)-gamma (γ) autoantibodies,” “anti-interferon (IFN)-gamma (γ) autoantibody,” “autoantibodies to Interferon (IFN)-gamma (γ),” “autoantibody to Interferon (IFN)-gamma (γ),” “interferon (IFN)-gamma (γ) autoantibody,” “interferon (IFN)-gamma (γ) autoantibodies,” “autoantibody against gamma (γ) interferon (IFN),” “autoantibodies against gamma (γ) interferon (IFN),” and “adult-onset immunodeficiency.” The references of the retrieved articles were reviewed for additional relevant citations. The abstracts of all identified articles were viewed, and the full-text versions of relevant articles were retrieved for data extraction and analysis. Decide whether a study met the inclusion criteria of this review and included in the study, which depended on two reviewers screened each record and each report retrieved. In addition, they worked independently. The systematic review protocol has not been registered in any database.

The inclusion criteria for articles and cases in the systematic literature review were as follows: 1) original case reports or cohort studies on AIGA published in English between January 1, 2004 and September 31, 2021; 2) articles describing the status of AIGA and exact infecting pathogen based on pathological and culture proof; and 3) patients with AIGA positivity who were repeated in the retrospective study (i.e., duplicate reports), cases’ data were extracted from the literature.

The exclusion criteria for articles and cases in the systematic literature review were as follows: 1) articles on diagnostics, immunology, experimental studies, and general review articles on AIGA; and 2) case reports or series of individuals with AIGA status not specified.

### Clinical outcomes definitions

The clinical course of infection was divided into the following four categories: 1) remission (complete or partial improvement of clinical symptoms after antimicrobial treatment and AIGA immunotherapy); 2) persistent infection (deterioration or no improvement of clinical symptoms after treatment); 3) relapsed infection (improvement of clinical symptoms, no pathogen detected after treatment, followed by the reappearance of pathogen-associated infectious signs and/or a positive pathogen test result); and 4) death.

### Data extraction

The following data were extracted: year of publication, geographical distribution, demographics, AIGA status, pathogens detected, immunotherapy for AIGA, and outcome. The geographical location was based on the corresponding author’s affiliations if the patient’s country and region were not specified in the article. If a case was reported in more than one publication, the most recent article was used for data extraction. The medical records in literature occurred were used for data extraction if a case described in our retrospective study again. In this process, two independently reviewers collected data from each report.

### Statistical analysis

Continuous variables are expressed as the median ± interquartile range. The primary objectives of this research were to study the epidemiology, geographical distribution, and pathogen spectrum of AIGA between 2004 and 2021. The secondary objectives included the analysis of immunotherapy and outcome of AIGA. The data were analyzed using IBM SPSS Statistics 27 (IBM Corp., Armonk, NY, USA) and GraphPad Prism version 7 (GraphPad Software Inc., San Diego, CA, USA).

## Results

### Demographics, epidemiology, and geographic characteristics of patients with AIGA positivity

**Figure 1:**
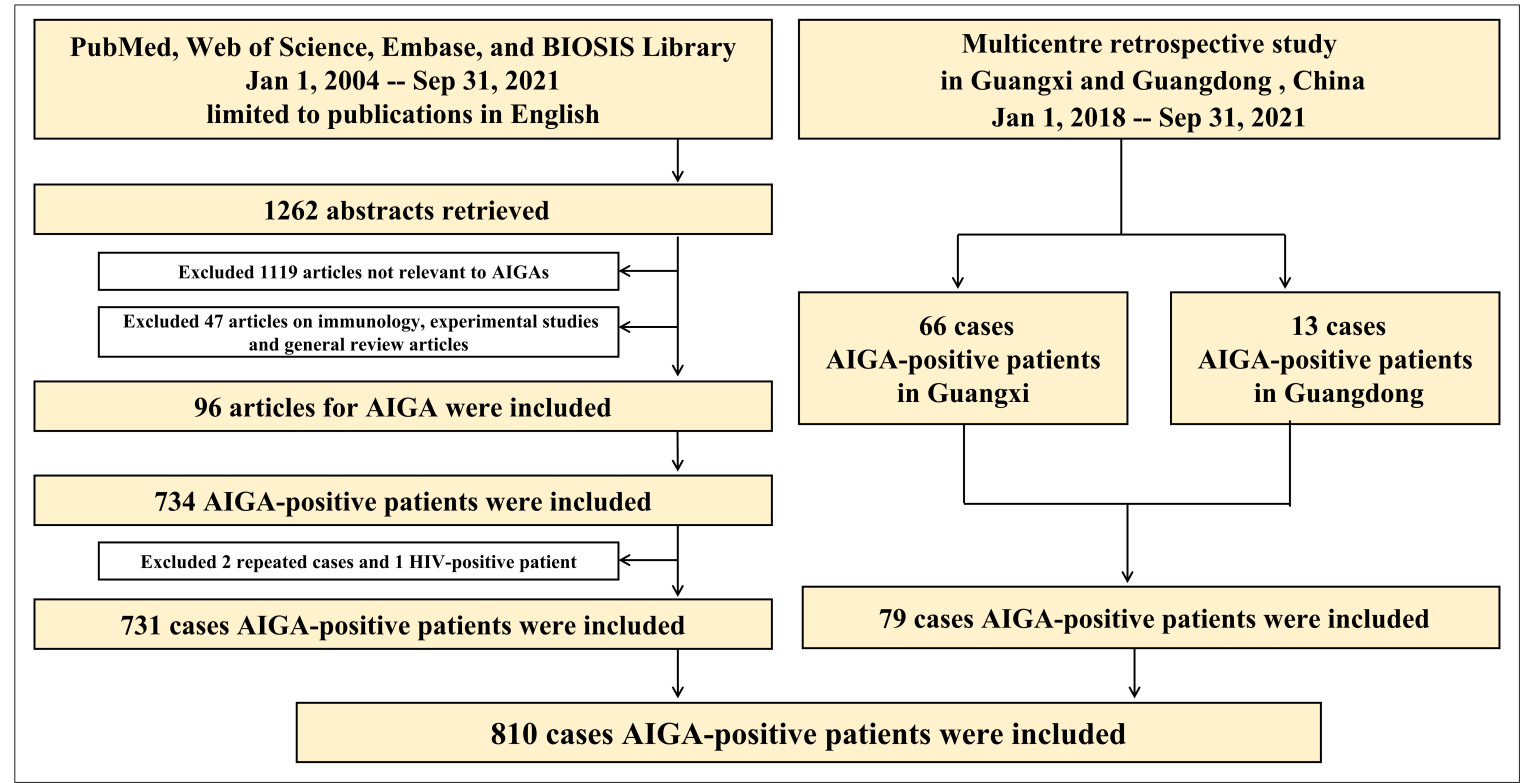
The literature search process and results of the systematic literature review and retrospective study. AIGA, anti-interferon-γ autoantibodies.

**Figure 2:**
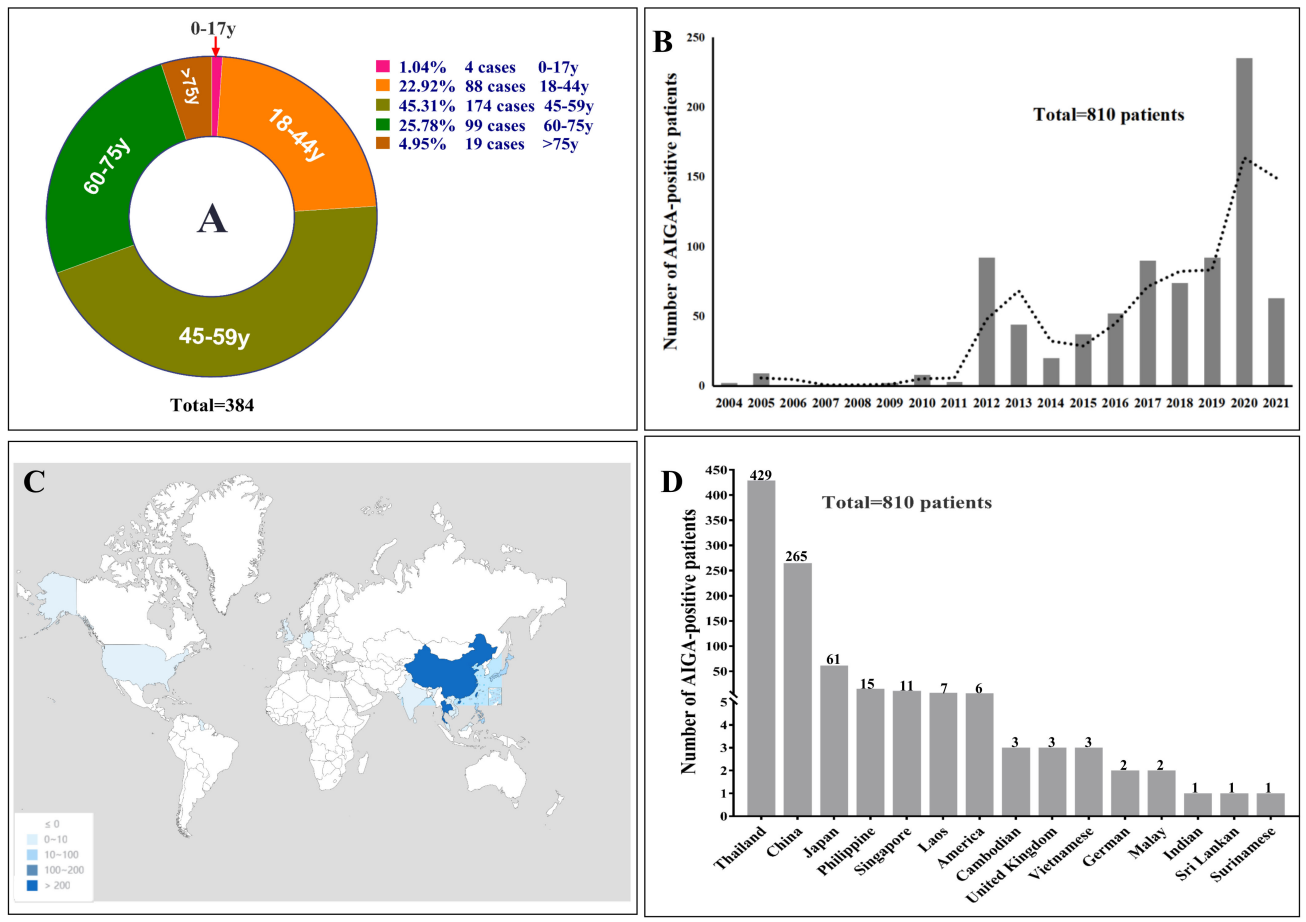
**(A)** The percentage of different age stages with AIGA-associated immunodeficiency. The reason for the statistical number of age, which was 384, was that most clinical cohort studies did not mention the age of each patient; therefore, the age data of the rest patients were missing in the systematic literature review. **(B)** annual incidence trends of AIGA. **(C, D)** geographical characteristics of the study participants.

Seventy-nine patients with AIGA positivity were enrolled in the retrospective study in Guangxi (66 cases) and Guangdong (13 cases). In the systematic literature review, 1262 articles were published in English between January 1, 2004 and September 31, 2021. After reviewing the article abstracts, 1119 articles were irrelevant to AIGA, and 47 on immunology, experimental studies, and general review articles were excluded. In addition, 96 articles were used as data sources (detailed information on the included articles are shown in **Supplementary Materials 1** and **2**). After excluding two duplicate cases in a retrospective study and one patient with HIV, 731 patients with AIGA positivity were identified in the systematic literature review. Thus, a total of 810 patients with AIGA positivity were included in the analysis (**Figure 1**). Among the included patients, age was reported in 384 patients, and sex was reported in 703 patients. The median age among the 384 patients was 52 (range, 10–87) years, and except for four teenagers, all patients were adults (aged >18 years) (**Figure 2A**). The age group with the highest incidence of AIGA positivity was the 45−59 years-old group (45.31%), followed by the 60−75 years-old group (25.78%) and the 18−44 years-old group (22.92%). Among the 703 patients, 47.08% (331/703) were men, and 52.92% (372/703) were women.

The reported incidence of AIGA-associated immunodeficiency syndrome has been increasing globally since 2012 (**Figure 2B**). The geographic distribution of AIGA in this study revealed an increased prevalence in Asia (798/810, 98.5%) (**Figure 2C** and **D**), particularly in Thailand (429/810, 53.0%) and China (267/810, 33.0%), overlapping with the geographical distribution of *Talaromyces marneffei*. In China, most AIGA cases were found in Guangxi (133/267, 49.8%) and Taiwan (103/267, 38.6%), followed by Hong Kong (13/267, 4.9%) and Guangdong (13/267, 4.9%), Zhejiang (2/267, 0.75%), Fujian (2/267, 0.75%) and Hunan (1/267, 0.37%).

### Pathogen spectrum of patients with AIGA positivity

**Figure 3:**
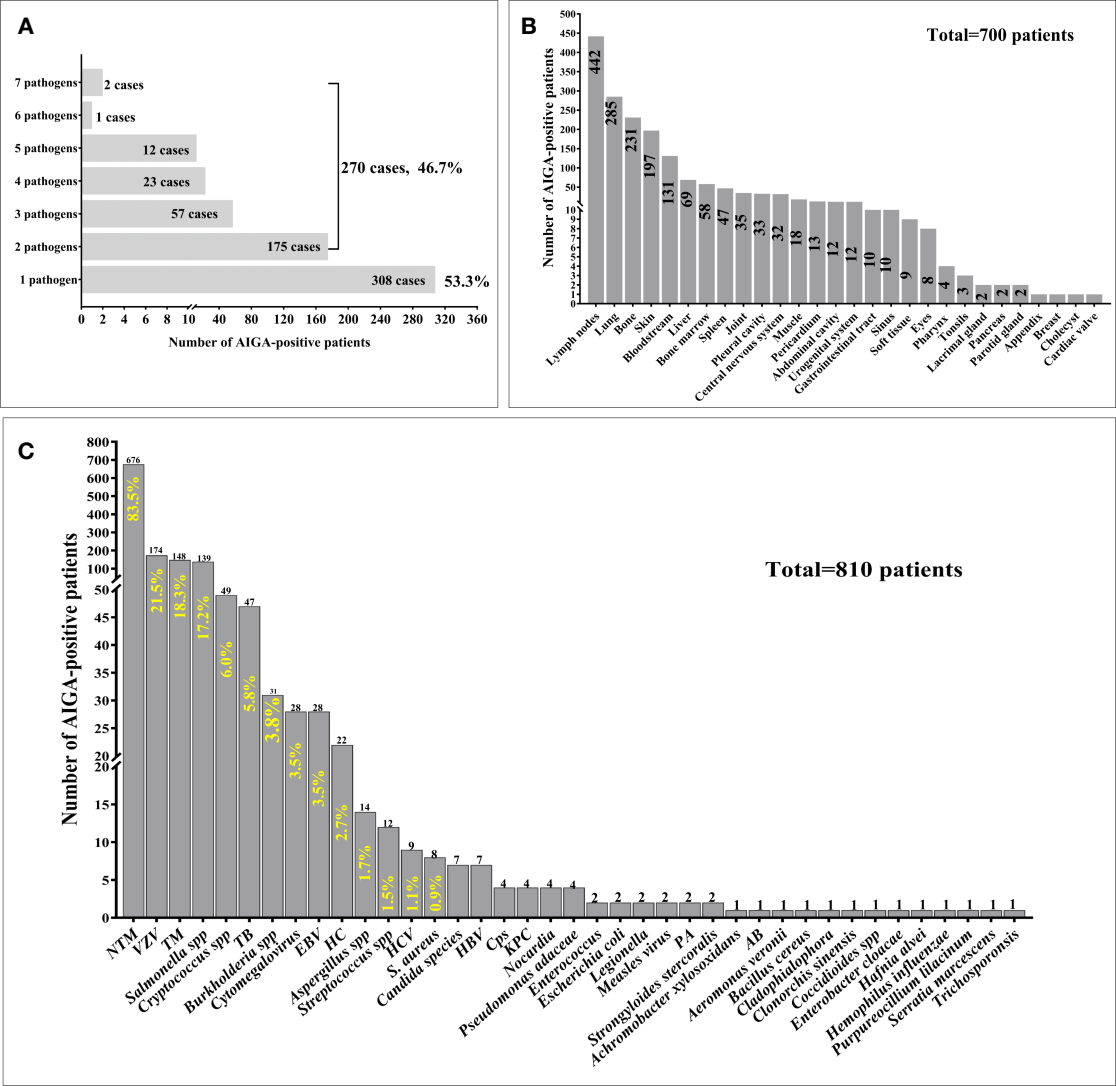
Pathogen spectrum and pathogen infection involvement sites of patients with anti-interferon-γ autoantibodies positivity. **(A)** Among 810 patients with AIGA positivity, 578 cases of pathogen infection were described in detail. 46.7% (270/578) of patients were simultaneously or successively infected with multiple pathogens. **(B)** Among 810 patients with AIGA positivity, 700 cases of pathogen infection involvement sites were described in detail. **(C)** Thirty-nine pathogens were found among the 810 patients with AIGA positivity. *S.Aureus*, *Staphylococcus aureus*, HC *Histoplasma capsulatum*; NTM, nontuberculous mycobacteria; TM,*Talaromyces marneffe*; TB *, Mycobacterium tuberculosis*; VZV*, Varicella-zoster virus*; EBV, *Epstein-Barr virus*; HBV, *Hepatitis B viru*s; HCV, *Hepatitis C virus*; Cps, *Chlamydophila psittaci*; KPC, *Klebsiella pneumoniae*; PA, *Pseudomonas aeruginosa*; AB, *Acinetobacter baumannii*.

Among 810 patients with AIGA positivity, 578 cases of pathogen infection were described in detail. Notably, 46.7% (270/578) of patients were simultaneously or successively infected with multiple pathogens. Some were infected with as many as seven pathogens (**Figure 3A**). The clinical characteristics and outcomes of the 79 patients with AIGA positivity, including 66 patients from Guangxi and 13 from Guangdong, are presented in **Supplementary Table 1**.

Among 810 patients with AIGA positivity, 700 cases of pathogen infection involvement sites were described in detail. The most common infection involvement sites were lymph nodes (63.14%), lung (40.71%), bone (33%), skin (28.14%), and bloodstream (18.71%), followed by liver (9.86%), bone marrow (8.26%), spleen (6.71%), and joint (5%) (**Figure 3B**).

Thirty-nine pathogens were identified among the 810 patients with AIGA positivity with one or more types of intracellular pathogen infection. The most common pathogens were NTM (83.5%), *Varicella-zoster virus* (21.5%), *Talaromyces marneffei* (18.3%), and *Salmonella* spp. (17.2%), followed by *Cryptococcus* spp., *Mycobacterium tuberculosis*, *Burkholderia* spp., C*ytomegalovirus*, *Epstein–Barr virus*, *Histoplasma capsulatum*, *Aspergillus* spp. (1.7%), and *Streptococcus spp* (**Figure 3A**).

### NTM infection spectrum in patients with AIGA positivity

**Figure 4:**
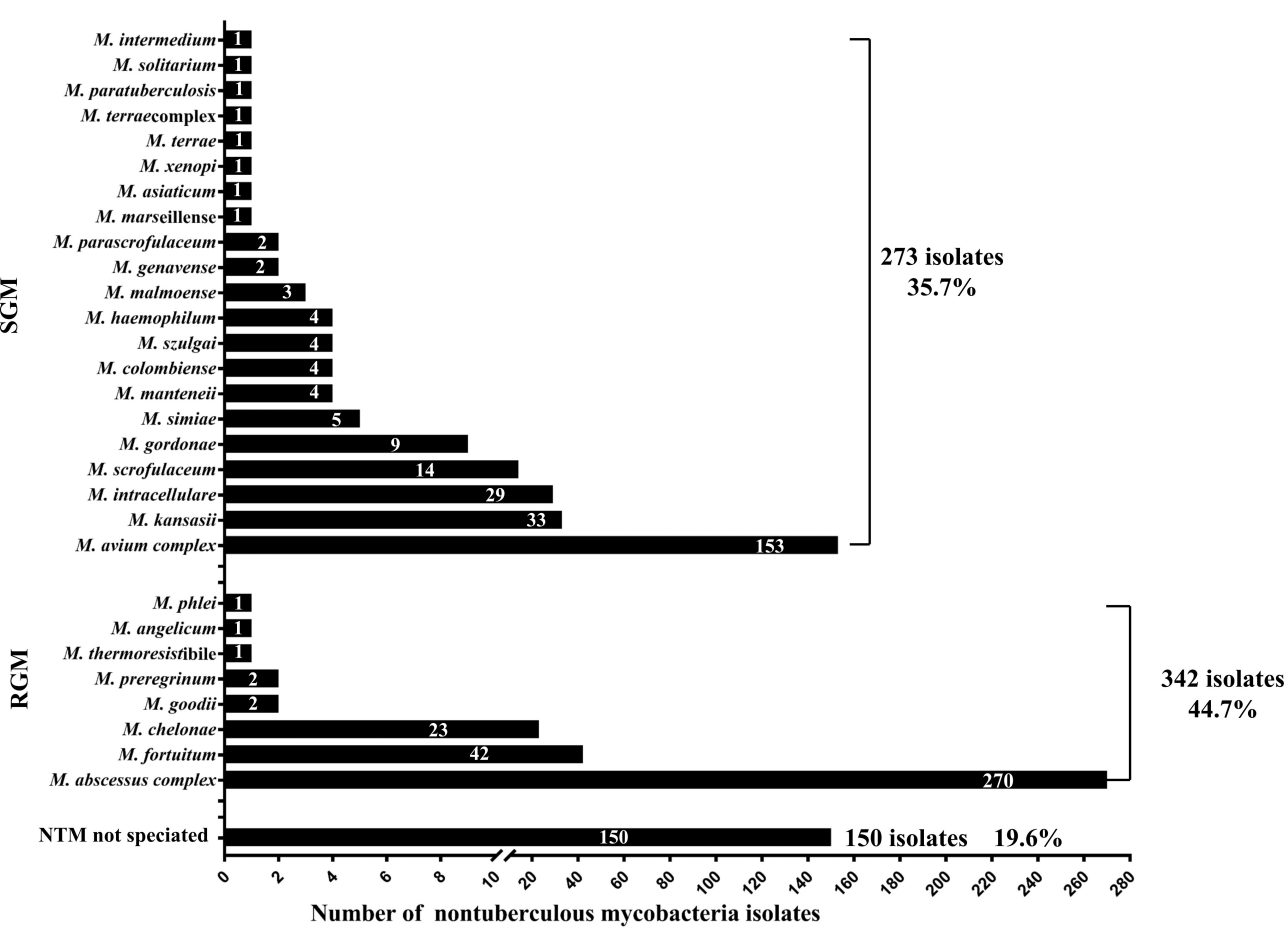
Nontuberculous mycobacteria isolates of patients with anti-interferon-γ autoantibodies positivity. Among the 810 patients, 676 were infected with NTM, and 765 NTM isolates were isolated from these 676 cases.

Among the 810 patients with AIGA positivity, 676 were infected with NTM. A total of 17.5% (118/676) of patients were simultaneously or successively infected with two or more NTM subtype infections. A total of 765 NTM isolates were identified from 676 cases of patients with NTM, including rapid-grower mycobacteria (44.7% of cases), slow-grower mycobacteria (35.7%), and unidentified NTM subtype (19.6%) (**Figure 4**). The most common subtypes of slow-grower mycobacteria were the *M. avium complex*, *M. kansasii*, *M. intracellulare*, and *M. scrofulaceum*. The most common subtypes of rapid-grower mycobacteria were *M. abscessus complex*, *M. fortuitum*, and *M. chelonae*.

### Attempt immunotherapy in patients with AIGA positivity

Among the 810 patients with AIGA positivity, outcomes of 495 patients were reported. Before the introduction of AIGA-related immunotherapy, the overall improvement rate was 45.1%, and >50% of patients could not achieve remission; there was a 12.3% mortality rate, 8.7% recurrence rate, and 33.9% persistent infection rate. In this study, 67 patients underwent immunotherapy for AIGA (**Supplementary Table 2**) (6, 7, 13–31), and the duration of anti-NTM or anti-fungal treatment in 29 patients was clearly described. The median duration of anti-NTM therapy was 15 (interquartile range: 7, 36) months, and in all of the 67 cases, infection was caused by persistent infection or deterioration after long-term regular antimicrobial therapy. Various immunotherapies have been used in AIGA, including 11 treatment options, which were small sample studies or case reports.

**Figure 5:**
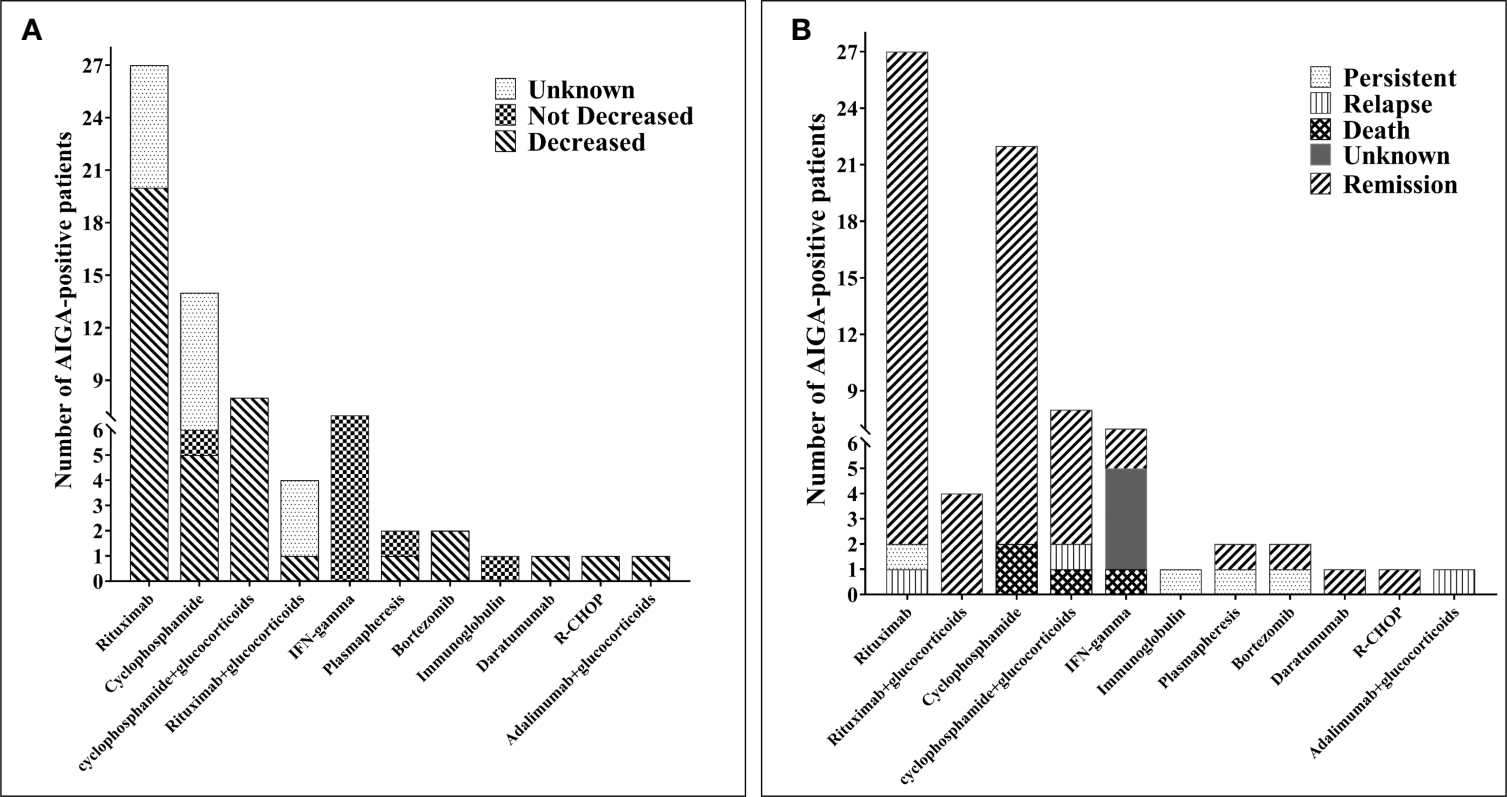
Immunotherapy of 67 patients with anti-interferon-γ autoantibodies positivity. **(A)** The number of changes in the AIGA titer of anti-interferon-γ autoantibodies positivity patients after immunotherapy. **(B)** The outcome of patients with anti-interferon-γ autoantibodies positivity after immunotherapy. CTX, cyclophosphamide; MP, methylprednisolone; RTX, rituximab.

The most commonly used immunotherapy strategies were rituximab (27/67, 40.3%) and cyclophosphamide (22/67, 32.8%), cyclophosphamide combined with glucocorticoids (8/67, 11.9%), recombinant human IFN-γ (7/67, 10.4%), rituximab combined with glucocorticoids (4/67, 6.0%), plasmapheresis (2/67, 3.0%), bortezomib (2/67, 3.0%), immunoglobulin (1/67, 1.5%), daratumumab (1/67, 1.5%), R-CHOP (1/67, 1.5%), and adalimumab combined with glucocorticoids (1/67, 1.5%) (**Figure 5A**). In 48 patients, AIGA titers decreased after immunotherapy, which included rituximab (20 cases), cyclophosphamide (13 cases), cyclophosphamide combined with glucocorticoid (eight cases), rituximab combined with glucocorticoid (one case), plasmapheresis (one case), bortezomib (two cases), daratumumab (one case), R-CHOP (one case), and adalimumab combined with glucocorticoid (one case) (**Figure 5B**). AIGA titer decreases were not observed in recombinant human IFN-γ and immunoglobulin. After AIGA treatment, only three patients died due to deterioration of infection, three had a recurrence, and one had persistent infections. The majority of the infected patients (60 cases) were in remission. Among these 67 patients, the duration of follow-up was clearly described in 42 cases, and only 25 patients were free of all antimicrobial agent use.

## Discussion

This systematic literature review and multicenter retrospective study tried to fill a gap in the knowledge regarding the epidemiological characteristics, incidence trends, pathogen spectrum, and possible effective immunotherapy in recent years of the emerging immunodeficiency diseases associated with AIGA positivity and provide deeper insight into anti-cytokine autoantibodies and guidance for clinical physicians.

During the study period, a remarkable increase in the incidence of patients with AIGA positivity was observed. This can be attributed to increased clinical awareness of AIGA and advancements in diagnostic methods and techniques (32). In present study, patients with AIGA positivity were mainly concentrated in Southeast Asia, particularly in Thailand and Southern China. Combined with previous studies, the concentration of the geographic distribution of AIGA in the population of Southeast Asia and Southern China suggests a possible genetic basis, the expression of specific HLA class II molecules, for the high incidence of AIGA-associated immune deficiency syndrome in these ethnic groups, particularly HLA class II alleles DRB1*16:02–DQB1*05:02 and HLA-DRB1*15:02–DQB1*05:01 (33).

Most AIGA-positive cases were adults, consistent with previous studies (6, 34). However, four teenagers (10−18 years of age) who were AIGA-positive with NTM infection (34), were also observed. Previous studies overemphasized the correlation between AIGA and NTM (8, 10), leading to neglect of other etiologies. In the present study, we characterized the complete pathogen spectrum of AIGA, including mycobacteria, fungi, bacteria, and viruses. Intracellular pathogens were found to be the most common infection and exhibited a continuously upward trend. The predominant phenotype associated with AIGA was disseminated NTM infection (82.5%) and disseminated *Talaromyces marneffei* infection (18.0%), with or without other opportunistic infections. A previous study found that 95% of non-HIV-infected patients with severe *Talaromyces marneffei* infection from Guangxi were AIGA positive (3). Both rapid growth mycobacteria (RGM) and slow growth mycobacteria (SGM) were found in AIGA, RGM (44.7%) was more common than SGM (35.7%), and there was an obvious RGM bias in patients with AIGA (RGM/SGM=1.25). *M. abscessus* was the most common type in RGM, and the *M. avium complex* was the most common type in SGM. Surprisingly, 46.7% of patients with AIGA positivity were infected with two or more pathogens successively or simultaneously and as many as seven pathogens successively. Therefore, patients should be screened for AIGA positivity when HIV-negative hosts have repeated infections with two or more intracellular pathogens.

In this study, even with long-term and intensive antimicrobial treatments, a large proportion of patients with AIGA positivity (42.6%) had recurrence and/or persistent infection. This may be because single antimicrobial therapy cannot reduce the titer of AIGA, leading to no improvement in the inhibition of IFN-γ signaling by AIGA, which was consistent with previous studies (6). Until now, there has been no unified understanding of whether AIGA is a primary immunodeficiency (PID) or autoimmune disease. In the 2022 Update of the International Union of Immunological Societies Phenotypical Classification for Human Inborn Errors of Immunity, AIGA was considered as a phenocopy of primary immunodeficiency, which was defined as adult-onset immunodeficiency with susceptibility to mycobacteria (1). However, obvious skin damage, particularly sweet syndrome, can be observed in patients with high AIGA titers, which supports possible autoimmune diseases (35). Therefore, immunotherapy, including corticosteroids, immunosuppressive drugs, and biological agents (rituximab), should be considered when the infection continues to worsen (6, 7, 13–31). However, these treatment options also affect protective immunity and often lead to serious side effects. To date, there is a lack of large sample data to evaluate the benefit of immunotherapy in AIGA patients, and the risk of complications and new infections caused by immunosuppressive and regulatory therapy is also unknown. Thus, we use these large data studies to fill the gap in knowledge regarding the optimal immunotherapeutic strategies for patients with AIGA positivity.

In this study, IFN-γ, immunoglobulin, and plasma exchange therapy were not effective, and thus not recommended. Rituximab and cyclophosphamide can effectively reduce AIGA titers, restore IFN-γ-mediated STAT1 phosphorylation function, and improve clinical symptoms and prognosis. The possible mechanism underlying rituximab treatment involves B-cell-targeting biologics for the treatment of immune-mediated diseases, direct against CD20, affect the B-cell population, and induce a decrease in the production of antibodies (36). Cyclophosphamide, a cytotoxic immunosuppressive agent, plays an immunosuppressive and anti-inflammatory role by blocking DNA synthesis, inhibiting cell proliferation, and by nonspecific killing of lymphocytes. The main effect of cyclophosphamide is to inhibit B-cell function; however, it also inhibits T-cell function, particularly regulatory T-cell function (37). Therefore, B-cell depletion was the most common side effect in this study. Small sample studies suggest that cyclophosphamide can reduce AIGA titers faster, with a longer remission time and lower incidence of recurrent infection than rituximab. However, some studies have reported that there is no significant difference in infection remission between these two regimens after 6 months of treatment (22). In addition, in some cases, an increase in AIGA titers was observed after rituximab and cyclophosphamide withdrawal. Therefore, it remains unclear which therapy is more effective; nonetheless, rituximab and cyclophosphamide can be used as immunotherapy to improve the prognosis of patients with AIGA positivity with poor response after long-term regular antimicrobial treatment. Notably, not all patients treated with rituximab or cyclophosphamide achieve the expected clinical response and reduced AIGA titer. In clinical practice, healthcare providers should consider turning to immunosuppressive drugs with other targets rather than extending the use of rituximab and cyclophosphamide. Recent case reports have identified other immunotherapies, such as daratumumab (a monoclonal antibody targeting CD38) (24), bortezomib (a proteosome inhibitor) (23), and adalimumab (a tumor necrosis factor inhibitor) (31), which can improve the prognosis of patients with a poor response to treatment with rituximab and cyclophosphamide.

## Conclusion

The trend analysis revealed a remarkably increasing trend in AIGA-related adult-onset immunodeficiency. These empirical findings provide a new understanding of AIGA and its pathogen spectrum, including not only NTM but also fungi, bacteria, and *Talaromyces marneffei*. Furthermore, dynamic observation of AIGA titer is valuable for monitoring disease activity. AIGA immunotherapy, including rituximab or cyclophosphamide, has yielded good preliminary results in some cases; however, the development of immunotherapy or alternative treatments for AIGA is urgently required, given the life-threatening nature of this condition. Further research is required to develop immunotherapy guidelines and elucidate the course of the disease, treatment side effects, and infection risk.

## Limitations

Some limitations of this research should be acknowledged. First, this is a systematic literature review and multicenter retrospective study conducted in Guangxi and Guangdong, China, and its conclusions may not apply to other countries and provinces. Second, in some patients, we did not assess the age, sex, treatment, and the change of AIGA titer. Third, although some new insights into the clinical characteristics of immunotherapy have been reported, the side effect and effectiveness of these treatment strategies remain unknown. However, our results comprehensively explored the epidemiological characteristics, incidence trends, pathogen spectrum, and possible effective immunotherapy in recent years. This study supports further research into the treatment strategies of AIGA in order to improve clinical practice. Furthermore, our next study will dissect the neutralizing mechanism of AIGA and the precise mechanisms underlying AIGA production.

## Data availability statement

The original contributions presented in the study are included in the article/supplementary material. Further inquiries can be directed to the corresponding authors.

## Ethics statement

The studies involving human participants were reviewed and approved by The Ethics Review Board of the First Affiliated Hospital of Guangxi Medical University approved the study(2018.KY-E-094). The patients/participants provided their written informed consent to participate in this study.

## Author contributions

All authors fulfilled the contribution requirements as per the International Committee of Medical Journal Editors role of authors and contributor guidelines. ZTL, JQZ and FY conceived and designed the study. YQ, WZ, GNF and FY carried out the analyses and wrote the first draft of the manuscript. MXT, YQ, XW, JLY and WZ carried out the patient recruitment and clinical sample collection. JQZ and ZTL contributed to the handling and testing of samples. FY and YQ contributed to the collection of data from the electronic medical records. All authors contributed to the article and approved the submitted version.
