# Supplementary material for: Pathogen spectrum and immunotherapy in patients with anti-IFN-γ autoantibodies: A multicenter retrospective study and systematic review
Source: Front Immunol. 2022 Dec 8;13:1051673. doi: 10.3389/fimmu.2022.1051673 (PMC9772057; doi:10.3389/fimmu.2022.1051673)
Supplement: Supplementary file 1 [file DataSheet_1.pdf]

ID	References	Patient	Age	Sex (M/F)	Geographic	Infection		AIGA titter	Diagnosis for AIGA positive	Neutralization activity	immunotherapy	AIGA titer change	Organs involvement	Outcome
1	Doffinger, 2004 [1]	1	47	M(1)	Philippines (1)	M.tuberculosis(1)<br>M.chelonae(1)<br>Candida spp(1)<br>CMV (1)	Rifampin, isoniazid, pyrazinamide, ethambutol for M.tuberculosis and cured. Ganciclovir for CMV. Isoniazid, pyrazinamide, ethambutol, amikacin, doxycycline, clarithromycin for M.chelonae. Persistent infection.	——	AIGA positive	Inhibited TNF-α production	Recombinant IFN-γ	No decrease	Lymph nodes（1）<br>Lung（1）<br>Liver（1）<br>Abdominal cavity（1）<br>Pericardium (1)<br>Spleen (1)<br>Pleural cavity (1)<br>Bone marrow(1)<br>Skin(1)	Death
2	Höflich C, 2004 [2]	1	25	F(1)	Thailand(1)	M. chelonae (1)<br>B. cocovenenans (1)<br>E. faecalis (1)<br>Candida spp. (1)	Antibiotic therapy for 6 months. B. cocovenenans was Relapse.<br>Deteriorated.	——	AIGA positive	Inhibited TNF-α production and HLA-DR expression.	None	——	Lymph nodes（1）<br>Lung（1）<br>Tonsils (1)<br>Spleen (1)<br>Liver（1）<br>Brain tissue (1)<br>Bone (1)<br>Bone marrow (1)<br>Skin (1)<br>Soft tissue (1)	Death
3	Kampmann B, 2005 [3]	3	46(1)	F(1)	United Kingdom(1)	M. avium intracellulare (1)	Rifabutin, clarithromycin, ethambutol, and clofazimine for M. avium intracellulare for 42 months. Deteriorate.	High ODs were detected (mean, 0.334)	AIGA positive	Inhibited TNF-α production	Recombinant IFN-γ	No decrease	Lymph nodes（1）<br>Joint(1)<br>Bone(1)<br>Soft tissue(1)<br>Lung(1)	Remission
			32(1)	M(1)	United Kingdom(1)	M. avium intracellulare (1)	Clarithromycin, rifabutin, and ethambutol. Improved.				None	——	Bone(1)	Remission
			59(1)	F(1)	United Kingdom(1)	M. fortuitum (1)<br>Aspergillus spp (1)<br>EBV(2)	Treatment with nontuberculous mycobacterial drugs and amphotericin.				None	——	Lung(1)	Remission
4	Patel SY, 2005[4]	6	43	F(1)	China	M. avium complex (1)	Treatment with anti-NTM drugs for 5 years	——	AIGA positive	——	None	——	Bone (1)<br>Lung (1)<br>Skin (1)<br>Soft tissue (1)<br>Knee (1)	Remission
			45	F(1)	Philippines (1)	M. avium complex (1)<br>M. chelonae (1)	Treatment with anti-NTM drugs more than 5 years	——	AIGA positive		None	——	Lymph nodes（1）<br>Lung (1)<br>Skin (1)	Persistent
			52	F(1)	Thailand(1)	M. tuberculosis (1)<br>M. szulgai (1)<br>M. kansasii (1)<br>M. scrofulaceum (1)	Treatment with anti-NTM drugs more than 5 years	——	AIGA positive		None	——	Lymph nodes（1）<br>Lung (1)<br>Skin (1)<br>Bone (1)	Persistent
			40	F(1)	Philippines (1)	M. abscessus (1)<br>M. fortuitum (1)<br>M. avium complex (1)	Treatment with anti-NTM drugs more than 5 years	——	AIGA positive		None	——	Lymph nodes（1）<br>Lung (1)	Persistent
			66	F(1)	Philippines (1)	M. abscessus (1)<br>M. avium (1)<br>HCV(1)<br>Pseudomonas aeruginosa (1)<br>Enterococcus cloacae (1)<br>Achromobacter xylosoxidans (1)	Treatment with anti-NTM drugs	——	AIGA positive		None	——	Lymph nodes（1）<br>Lung (1)	Persistent
			31	F(1)	Philippines (1)	M. avium complex (1)<br>VZV (1)	Treatment with anti-NTM drugs more than 2.5 years	——	AIGA positive		None	——	Appendix (1)<br>Bone (1)<br>Skin (1)<br>Pharynx (1)	Persistent
5	Tanaka Y, 2007[5]	1	54	M(1)	Japan (1)	M. avium complex (1)<br>Streptococcus pyogene (1)	Sulbactam/ampicillin for Streptococcus pyogene Clarithromycin, ethambutol, streptomycin for M. avium complex	——	AIGA positive	Inhibited IFN-γ production	None	——	Lung (1)<br>Bone (1)<br>Lymph nodes（1）<br>Gastrointestinal tract (1)<br>Bone marrow (1)<br>Pleural cavity (1)<br>Spleen (1)<br>Liver（1）<br>Abdominal cavity（1）	Remission
6	Koya T, 2009 [6]	1	44	F(1)	Japan (1)	M. avium complex (1)	Rifampicin, streptomycin, ethambutol, and clarithromycin for M. avium complex. Deteriorated.	——	AIGA positive	——	Intravenous mmunoglobulin	No decrease	Soft tissue (1)<br>Bone (1)	Remission
7	Baerlecken N, 2009 [7]	1	38	F(1)	Philippines (1)	M. avium intercellulare (1)	Rifabutin, ethambutol, and azithromycin	——	AIGA positive	Inhibited IFN γ production and MHC-I expression	Plasmapheresis<br>Cyclophosphamide<br>Prednisolone	Decrease	Lung (1)<br>Bone (1)<br>Liver (1)<br>Spine (1)<br>Blood (1)	Remission

8	Tang BS, 2010 [8]	8	42	F(1)	China (1)	M. chelonae (1)<br>M. kansasii (1)<br>T. marneffei (1)<br>B. pseudomallei (1)	Imipenem, amikacin and tigecycline for NTM. Relapse. Itraconazole for T. marneffei,	OD value: 2.649	AIGA positive	——	None	——	Lymph nodes（1）<br>Liver (1)<br>Spleen (1)<br>Joint (1)	Relapse
			45	F(1)	China (1)	T. marneffei (1)<br>M. avium intercellulare (1)<br>M. fortuitum (1)<br>HBV (1)	Isoniazid, ethambutol, clarithromycin, ofloxacin, amikacin and<br>levofloxacin for M. fortuitum<br>Amphotericin B, itraconazole for T. marneffei .	OD value: 2.814	AIGA positive	——	None	——	Lung (1)<br>Lymph nodes（1）<br>Bone (1)<br>Bone marrow (1)	Relapse
			39	F(1)	China (1)	T. marneffei (1)<br>M. kansasii (1)<br>Salmonella enterica (1)	Cefoperazone/sulbactam, ampicillin for Salmonella enterica.<br>Azithromycin, rifampin, and levofloxacin for M. kansasii	OD value: 1.319	AIGA positive	——	None	——	Lung (1)<br>Lymph nodes（1）<br>Bone (1)	Not mentioned
			67	F(1)	China (1)	T. marneffei (1)<br>M. avium intercellulare (1)<br>B. pseudomallei (1)	——	OD value: 2.809	AIGA positive	——	None	——	Bone (1)<br>Bone marrow (1)	Not mentioned
			53	M(1)	China (1)	T. marneffei (1)<br>M. chelonae (1)<br>Non-typhoidal salmonellosis (1)<br>B. pseudomallei (1)	——	OD value: 2.593	AIGA positive	——	None	——	Lymph nodes（1）	Not mentioned
			49	M(1)	China (1)	T. marneffei (1)<br>Salmonella spp (1)<br>B. pseudomallei (1)	——	OD value: 2.9	AIGA positive	——	None	——	Lung (1)<br>Lymph nodes（1）	Not mentioned
			87	M(1)	China (1)	T. marneffei (1)<br>M. avium intercellulare (1)<br>Salmonella spp (1)<br>B. pseudomallei (1)<br>HBV (1)	——	OD value: 0.959	AIGA positive	——	None	——	Bone (1)<br>Lymph nodes（1）	Not mentioned
			54	M(1)	China (1)	T. marneffei (1)<br>M. chelonae (1)<br>Salmonella spp (1)<br>HBV (1)	——	OD value: 1.358	AIGA positive	——	None	——	Lymph nodes（1）<br>Pericardial (1)<br>Pleural cavity (1)	Not mentioned
9	Kampitak T, 2011 [9]	3	56	M(1)	Thailand(1)	T. marneffei (1)<br>M. intermedium (1)<br>M. tuberculosis (1)	Isoniazid, rifampin, pyrazinamide, Moxifloxacin and ethambutol for M. intermedium. Itraconazole for T. marneffei.	Positive (high titer)	AIGA positive	Inhibitory activity against IFN-γ	None	——	Lymph nodes（1）<br>Lung (1)<br>Skin (1)<br>Bone marrow (1)	Relapse
			45	M(1)	Thailand(1)	Salmonella spp (1)<br>M. tuberculosis (1)<br>VZV (1)<br>M.abscessus (1)	Ceftriaxone, isoniazid, rifampin, pyrazinamide and ethambutol for tuberculosis and salmonellosis. Salmonellosis was relapse.	Positive (high titer)	AIGA positive	Inhibitory activity against IFN-γ	None	——	Lymph nodes（1）<br>Lung (1)<br>Skin (1)<br>Bone marrow (1)<br>Pleural cavity (1)<br>Liver (1)	Relapse
			39	M(1)	Thailand(1)	Salmonella spp (1)<br>M. abscessus (1)<br>M. tuberculosis (1)	Isoniazid, rifampicin, pyrazinamide, ethambutol and clarithromycin for TB and NTM infection, ciprofloxacin for salmonellosis.	Positive (high titer)	AIGA positive	Inhibitory activity against IFN-γ	None	——	Lymph nodes（1）<br>Skin (1)<br>Bone (1)<br>Abdomen (1)	Persistent
10	Browne SK， 2012 [10]	78	—	—	Thailand<br>China	——	——	——	——	——	——	——	——	——
11	Browne SK， 2012 [11]	4	46(1)	F(1)	Philippines (1)	M. abscessus (1)<br>M. avium complex (1)	Clarithromycin, ethambutol, ioniazid, linezolid, moxifloxacin, tigecycline for anti-NTM, Relapsed twice	——	AIGA positive	Inhibited pSTAT-1	Rituximab	80% decrease	Lymph nodes（1）<br>Skin (1)<br>Blood (1)<br>Urine (1)<br>Pelvic (1)	Remission
			69	F(1)	Philippines (1)	M. abscessus (1)	Amikacin, amoxicillin/clavulanate, azithromycin, ciprofloxacin, ertapenem, ethambutol, isoniazid, linezolid, meropenem, pyrazinamide, rifampin, tigecycline for NTM. Relapsed twice	——	AIGA positive	Inhibited pSTAT-1	Rituximab	73.7% decrease	Lymph nodes（1）<br>Blood (1)<br>Bone (1)	Remission
			50	F(1)	Laos (1)	M. avium (1)	Clarithromycin, Ethambutol, Moxifloxacin for anti-NTM, Relapsed twice.	——	AIGA positive	Inhibited pSTAT-1	Rituximab	65% decrease	Lymph nodes（1）<br>Muscle (1)<br>Bone (1)	Remission
			60	F(1)	Vietnam (1)	M. intracellulare (1)	Amikacin, azithromycin, clarithromycin, ethambutol, isoniazid, levofloxacin, moxifloxacin, pyrazinamide, rifampin for NTM.	——	AIGA positive	Inhibited pSTAT-1	Rituximab	Decrease 58%	Skin (1)<br>Muscle (1)<br>Bone (1)	Remission
12	Picque JB, 2012 [12]	1	45	F(1)	Thailand (1)	M. scrofulaceum (1)<br>M. avium (1)<br>T. marneffei (1)	Amphotericin B and Itraconazole for T. marneffei. Azithromycin, rifambutin, ethambutol, moxifloxacin, amikacin for M. scrofulaceum.	——	AIGA positive	——	None	——	Lymph nodes (1)<br>Lung (1)<br>Bone (1)<br>Blood (1)<br>Pleural cavity (1)	Persistent
13	Tanaka H, 2013[13]	1	64	F(1)	Japan (1)	M. avium (1)<br>Mycobac terium intracellulare (1)<br>Measles virus (1)<br>VZV (1)	Clarithromycin, rifampicin, and ethambutol for NTM.	10,000-fold titer of anti-IFN-γ antibody compared with controls and other disease groups.	AIGA positive	——	None	——	Bone (1)<br>Lung (1)<br>Skin (1)<br>Urinary bladder (1)<br>Uterus (1)	Relapse

14	Nei T, 2012 [14]	1	53	M(1)	Japan (1)	M. kansasii (1)	Isoniazid, rifampicin and ethambutol for M. kansasii	——	AIGA positive	Inhibited TNF-α production	None	——	Bone (1)<br>Lung (1)<br>Lymph nodes (1)<br>Liver (1)<br>Spleen (1)<br>Bone marrow (1)	Death
15	Chi CY, 2013 [15]	17	55	M(1)	China (1)	M. abscessus (1)<br>S. enteritidis (1)<br>T. marneffei (1)<br>Salmonella spp (1)<br>VZV (1)	——	——	AIGA positive	Inhibited IL-12 production	None	——	Lymph nodes (1)<br>Bone (1)	Remission
			51	F(1)	China (1)	M. scrofulaceum (1)<br>VZV (1)	——	——	AIGA positive	Inhibited IL-12 production	None	——	Lymph nodes (1)<br>Bone (1)<br>Skin (1)	Persistent
			69	M(1)	China (1)	M. avium complex (1)<br>S enteritidis (1)<br>Salmonella spp (1)<br>VZV (1)	——	——	AIGA positive	Inhibited IL-12 production	None	——	Lymph nodes (1)<br>Bone(1)	Persistent
			47	F(1)	China (1)	S enteritidis (1)<br>M. gordonae (1)<br>Salmonella spp (1)<br>VZV (1)	——	——	AIGA positive	Inhibited IL-12 production	None	——	Lymph nodes (1)<br>Bone(1)	Persistent
			55	M(1)	China (1)	M. terrae (1)	——	——	AIGA positive	Inhibited IL-12 production	None	——	Muscles (1)<br>Bone (1)	Remission
			81	M(1)	China (1)	NTM(1)	——	——	AIGA positive	Inhibited IL-12 production	None	——	Lymph nodes (1)<br>Skin (1)	Persistent
			66	M(1)	China (1)	NTM(1)	——	——	AIGA positive	Inhibited IL-12 production	None	——	Lymph nodes (1)<br>Lung(1)	Remission
			87	M(1)	China (1)	M. scrofulaceum(1)<br>S aureus (1)<br>VZV (1)	——	——	AIGA positive	Inhibited IL-12 production	None	——	Lymph nodes (1)<br>Skin (1)<br>Bone (1)	Remission
			48	M(1)	China (1)	M. avium complex (1)<br>S enteritidis (1)<br>VZV (1)	——	——	AIGA positive	Inhibited IL-12 production	None	——	Lymph nodes (1)<br>Lung (1)<br>Bone (1)	Remission
			61	F(1)	China (1)	NTM (1)<br>VZV (1)	——	——	AIGA positive	Inhibited IL-12 production	None	——	Lymph nodes (1)<br>Skin(1)<br>Bone (1)	Persistent
			49	M(1)	China (1)	M. kansasii (1)<br>VZV (1)	——	——	AIGA positive	Inhibited IL-12 production	None	——	Lymph nodes (1)<br>Lung (1)	Persistent
			56	F(1)	China (1)	NTM (1)<br>VZV (1)	——	——	AIGA positive	Inhibited IL-12 production	None	——	Lymph nodes (1)<br>Skin (1)<br>Bone (1)	Persistent
			46	F(1)	China (1)	NTM (1)<br>S. enteritidis (1)	——	——	AIGA positive	Inhibited IL-12 production	None	——	Lymph nodes (1)<br>Lung (1)	Persistent
			72	F(1)	China (1)	M. avium complex(1)	——	——	AIGA positive	Inhibited IL-12 production	None	——	Bone (1)	Death
			49	F(1)	China (1)	NTM (1)<br>Legionellosis (1)<br>T. marneffei(1)	——	——	AIGA positive	Inhibited IL-12 production	None	——	Lung (1)<br>Lymph nodes (1)<br>Pericardium (1)<br>Bone (1)	Lost
			47	F(1)	China (1)	NTM (1)<br>S. enteritidis(1)	——	——	AIGA positive	Inhibited IL-12 production	None	——	Lung(1)<br>Lymph nodes (1)<br>Skin (1)<br>Bone (1)	Persistent
			76	M(1)	China (1)	M. abscessus (1)<br>M. chelonae (1)	——	——	AIGA positive	Inhibited IL-12 production	None	——	Lung (1)<br>Lymph nodes (1)	Persistent
16	Wongkulab P, 2013 [16]	20	mean 48	F(11)<br>M(9)	Thailand(20)	M. chelonae(3)<br>M. abscessus(4)<br>M. chelonae-abscessus complex (3)<br>M. fortuitum (1)<br>M. avium (3)<br>M. avium/paratuberculosis/solitarium (1)<br>M. kansassii (3)<br>NTM Species not specified (6)<br>T. marneffei (12)<br>Cryptococcosis(1)<br>Histoplasmosis (1)<br>Salmonella spp (8)	——	Mean concentrations of autoantibody to IFN-γ were 2.4606±1.309 O.D.	AIGA positive	Inhibitory activity against IFN-γ	None	——	Lymph nodes (15)<br>Skin (14)<br>Blood (11)<br>Lung(5)<br>Bone and joint (4)<br>Others (2)	Not mentioned
17	Ishii T， 2013 [17]	1	66	M(1)	Japan (1)	M. aviumcomplex (1)<br>HCV (1)	Rifampicin, ethambutol, clarithromycin, amikacin, levofloxacin hydrate, moxifloxacin for M. aviumcomplex.	——	AIGA positive	——	None	——	Lung(1)<br>Lymph nodes (1)<br>Bone (1)	Remission

18	Poulin S, 2013 [18]	1	49	F(1)	Laos(1)	M. colombiense (1)<br>M. cytomegalovirus (1)<br>P. aeruginosa (1)	——	——	AIGA positive	——	None	——	Lung(1)<br>Lymph nodes (1)	Death
19	Chan JF, 2013 [19]	3	57	M(1)	China (1)	M. chelonae (1)<br>T. marneffei (1)<br>Salmonella spp(1)<br>VZV (1)	Azithromycin, ceftazidime, vancomycin, itraconazole, imipenem/cilastatin, clarithromycin	——	AIGA positive	——	None	——	Skin(1)<br>Lymph nodes (1)	Remission
			42	F(1)	China (1)	M. chelonae (1)<br>M. Kansasii (1)<br>T. marneffei (1)<br>Burkholderia pseudomallei(1)	Imipenem/cilastatin, amikacin and clarithromycin	——	AIGA positive	——	None	——	Skin(1)<br>Lymph nodes (1)<br>Spleen (1)<br>Joint (1)	Remission
			47	F(1)	China (1)	M. chelonae (1)<br>VZV (1)	Imipenem/cilastatin, amikacin and clarithromycin	——	AIGA positive	——	None	——	Skin(1)<br>Lymph nodes (1)	Relapse
20	Chan JF, 2013 [20]	1	34	F(1)	China (1)	M. chelonae (1)<br>HBV (1)	Imipenem-cilastatin, amikacin, clarithromycin	——	AIGA positive	——	None	——	Skin(1)<br>Lymph nodes (1)<br>Lung (1)	Remission
21	Chetchotisakd P, 2014 [21]	16	53	F(1)	Thailand(1)	Slow grower NTM	Isoniazid, regimen, ethambutol, clarithromycin, ofloxacin, amikacin, azithromycin, linezolid	——	Not detected	——	None	——	Skin(1)<br>Lymph nodes (1)<br>Spleen (1)<br>Lung (1)<br>Liver (1)<br>Blood (1)	Death
			47	F(1)	Thailand(1)	Cryptococcosis (1)<br>M. abscessus(1)	Ethambutol, clarithromycin, rifampicin, imipenem, linezolid	——	AIGA positive	——	None	——	Lymph nodes (1)<br>Bone (1)	Relapse
			61	M(1)	Thailand(1)	M. abscessus(1)	Clarithromycin, amikacin, ciprofloxacin, linezolid	——	AIGA positive	——	None	——	Lymph nodes (1)	Remission
			15	F(1)	Thailand(1)	VZV (1)<br>M. fortuitum(1)	Linezolid, isoniazid, rifampicin, ethambutol, clarithromycin, ofloxacin, azithromycin, amikacin,	——	AIGA positive	——	None	——	Lymph nodes (1)<br>Skin(1)	Remission
			47	F(1)	Thailand(1)	M. preregrinum(1)	Linezolid, isoniazid, rifampicin, ethambutol, ofloxacin, azithromycin	——	AIGA positive	——	None	——	Lymph nodes (1)<br>Lung (1)	Relapse
			48	M(1)	Thailand(1)	VZV (1)<br>M. abscessus (1)	Linezolid, isoniazid, rifampicin, ethambutol, ofloxacin, clarithromycin, azithromycin, amikacin, imipenem	——	AIGA positive	——	None	——	Skin(1)<br>Lymph nodes (1)<br>Abdominal (1)<br>Liver (1)<br>Lacrimal gland (1)	Persistent
			45	F(1)	Thailand(1)	VZV (1)<br>M. abscessus (1)	Linezolid, clarithromycin, amikacin, imipenem, doxycycline, ofloxacin	——	AIGA positive	——	None	——	Lymph nodes (1)<br>Skin(1)<br>Bone (1)	Relapse
			43	F(1)	Thailand(1)	Cryptococcosis (1)<br>VZV (1)<br>M. abscessus(1)	Linezolid, ethambutol, clarithromycin, amikacin, ofloxacin, isoniazid,imipenem	——	AIGA positive	——	None	——	Lymph nodes (1)<br>Liver(1)<br>Bone (1)	Relapse
			42	F(1)	Thailand(1)	Salmonella spp(1)<br>M. tuberculosis (1)<br>VZV (1)<br>Cladophialophora (1)<br>Rapid grower NTM	Ofloxacin, azithromycin, imipenem, clarithromycin, moxifloxacin, linezolid	——	AIGA positive	——	None	——	Lymph nodes (1)<br>Liver(1)<br>Spleen (1)<br>Lacrimal gland (1)	Death
			47	F(1)	Thailand(1)	VZV (1)<br>M. simiae (1)<br>M. abscessus (1)<br>M. fortuitum (1)	Linezolid, clarithromycin, ofloxacin, ethambutol, isoniazid, rifampicin, ciprofloxacin	——	AIGA positive	——	None	——	Lymph nodes (1)<br>Skin(1)<br>Sinus (1)<br>Tonsil (1)	Remission
			45	F(1)	Thailand(1)	VZV (1)<br>M. fortuitum (1)	Linezolid, ethambutol, clarithromycin, ofloxacin	——	AIGA positive	——	None	——	Lymph nodes (1)	Persistent
			57	F(1)	Thailand(1)	Salmonella spp(1)<br>VZV (1)<br>M. abscessus (1)	Linezolid, clarithromycin, amikacin, imipenem, doxycycline, ofloxacin	——	AIGA positive	——	None	——	Lymph nodes (1)<br>Liver(1)<br>Spleen (1)<br>Blood (1)	Persistent
			48	F(1)	Thailand(1)	Cryptpcoccosis (1)<br>M. abscessus (1)	Linezolid, clarithromycin, ofloxacin, ethambutol, imipenem, cefoxitin, amikacin	——	AIGA positive	——	None	——	Lymph nodes (1)<br>Bone (1)	Persistent
			63	M(1)	Thailand(1)	Histoplasmosis (1)<br>VZV (1)<br>M. abscessus (1)	Linezolid, clarithromycin, ofloxacin, imipenem	——	AIGA positive	——	None	——	Lymph nodes (1)<br>Liver(1)<br>Bone (1)	Remission
			34	M(1)	Thailand(1)	Cryptococcosis (1)<br>M. tuberculosis (1)<br>M. abscessus (1)	Linezolid, azithromycin, ofloxacin, imipenem	——	AIGA positive	——	None	——	Lymph nodes (1)	Persistent
			34	F(1)	Thailand(1)	VZV (1)<br>M. abscessus (1)	Linezolid, isoniazid, clarithromycin, ofloxacin, ethambutol,azithromycin, imipenem	——	AIGA positive	——	None	——	Lymph nodes (1)	Remission
22	DeLeon TT, 2014[22]	1	77	M(1)	Philippines (1)	Salmonella spp(1)<br>M. Avium Complex(1)	Treatment with antimicrobial agents	——	AIGA positive	——	None	——	Lung (1)<br>Thoracic cavity (1)<br>Digestive system (1)	Remission
23	Suzuki K, 2014[23]	1	74	F(1)	Japan (1)	M. fortuitum (1)	Levofloxacin, imipenem/cilastatin, amikacin, clarithromycin	54 E.U.<br>(control 18 E.U.)	AIGA positive	Inhibited STAT-1 phosphorylation (STAT1 phosphorylation index: 47, control 407)	None	——	Lymph nodes (1)<br>Lung (1)	Remission

24	Czaja CA, 2014 [24]	1	78	M(1)	Japan (1)	EBV (1)<br>M. chelonae–abscessus (1)<br>VZV(1)<br>Cytomegalovirus(1)	Azithromycin, imipenem/cilastatin, and tobramycin.Recurrences twice.	——	AIGA positive	——	Rituximab	——	Lymph nodes (1)<br>Lung (1)<br>Joint (1)<br>Bone (1)<br>Skin (1)	Remission
25	O'Connell E, 2014 [25]	1	39	F(1)	America(1)	M. aviumcomplex (1)	Isoniazid, rifampin, ethambutol, pyrazinamide, azithromycin.<br>Persistent	——	AIGA positive	Inhibited pSTAT-1 and IL-12 production	None	——	Throat (1)<br>Brain(1)<br>Lymph nodes (1)<br>Lung (1)	Persistent
26	Hase I, 2015 [26]	1	65	M(1)	Japan (1)	M. gordonae (1)<br>M. mantenii (1)	Clarithromycin, rifampicin, ethambutol	——	AIGA positive	——	None	Decreased gradually with the above-mentioned treatment, though it remained above the normal level.	Bone (1)<br>Lung (1)<br>Lymph nodes (1)<br>Skin (1)	Remission
27	Otome O, 2015 [27]	1	71		Cambodia(1)	M. haemophilum(1)	Clarithromycin, ciprofloxacin, rifampicin	——	AIGA positive	——	None	——	Bone (1)<br>Lung (1)<br>Thoracic cavity (1)	Remission
28	Pithukpakorn M,	32	43	F(1)	Thailand(1)	M. tuberculosis (1)<br>Salmonella spp(1)<br>M.abscessus (1)	——	——	AIGA positive	——	None	——	Blood (1)<br>Lung (1)	Remission
			57	F(1)	Thailand(1)	Histoplasma capsulatum(1)<br>T. marneffei (1)<br>Burkholderia pseudomallei(1)<br>M.abscessus(1)<br>Salmonella spp(1)	——	——	AIGA positive	——	None	——	Lymph nodes (1)<br>Joint (1)	Death
			53	M(1)	Thailand(1)	M.abscessus (1)<br>M.intracellulare (1)<br>VZV (1)	——	——	AIGA positive	——	None	——	Lymph nodes (1)<br>Blood (1)<br>Lung (1)	Remission
			37	F(1)	Thailand(1)	M.abscessus (1)<br>Salmonella spp(1)	——	——	AIGA positive	——	None	——	Lymph nodes (1)<br>Blood (1)	Death
			57	F(1)	Thailand(1)	M.abscessus (1)	——	——	AIGA positive	——	None	——	Lymph nodes (1)	Remission
			48	F(1)	Thailand(1)	Salmonella spp(1)	——	——	AIGA positive	——	None	——	Blood (1)	Remission
			48	F(1)	Thailand(1)	M.abscessus(1)	——	——	AIGA positive	——	None	——	Bone marrow (1)	Remission
			69	M(1)	Thailand(1)	Salmonella spp(1)<br>M.hemophilum(1)	——	——	AIGA positive	——	None	——	Bone marrow (1)<br>Blood (1)	Remission
			57	F(1)	Thailand(1)	M.abscessus(1)	——	——	AIGA positive	——	None	——	Lymph nodes (1)<br>Blood (1)	Remission
			60	F(1)	Thailand(1)	M.fortuitum (1)<br>VZV (1)<br>T. marneffei (1)<br>M. abscessus (1)	——	——	AIGA positive	——	None	——	Lymph nodes (1)<br>Blood (1)<br>Skin (1)	Remission
			26	M(1)	Thailand(1)	M.abscessus<br>Cryptococcus neoformans<br>M.fortuitum<br>T. marneffei (1)	——	——	AIGA positive	——	None	——	Lymph nodes (1)<br>Blood (1)<br>Skin (1)<br>Cerebrospinal fluid (1)<br>Jiont (1)<br>Skin soft tissue (1)	Death
			51	M(1)	Thailand(1)	Salmonella spp (1)<br>M.abscessus (1)<br>VZV (1)	——	——	AIGA positive	——	None	——	Lymph nodes (1)<br>Blood (1)	Remission
			68	M(1)	Thailand(1)	M.abscessus (1)<br>VZV (1)	——	——	AIGA positive	——	None	——	Lymph nodes (1)	Remission
			57	F(1)	Thailand(1)	M.abscessus (1)<br>Salmonella spp (1)<br>Hemophilus influenzae(1)	——	——	AIGA positive	——	None	——	Lymph nodes (1)<br>Blood (1)<br>Skin (1)<br>Lung (1)	Death
			24	M(1)	Thailand(1)	M.abscessus(1)	——	——	AIGA positive	——	None	——	Lymph nodes (1)<br>Blood (1)<br>Lung (1)	Remission
			74	M(1)	Thailand(1)	M.scrofulaceum (1)<br>M.fortuitum(1)	——	——	AIGA positive	——	None	——	Lymph nodes (1)<br>Skin (1)	Remission
			46	F(1)	Thailand(1)	M.aviumcomplex (1)<br>VZV (1)	——	——	AIGA positive	——	None	——	Lymph nodes (1)	Remission
			59	M(1)	Thailand(1)	M.abscessus (1)	——	——	AIGA positive	——	None	——	Lymph nodes (1)	Remission
			33	F(1)	Thailand(1)	Salmonella spp (1)<br>VZV (1)<br>M.abscessus (1)	——	——	AIGA positive	——	None	——	Lymph nodes (1)<br>Blood (1)	Persistent
			47	M(1)	Thailand(1)	Burkholderia pseudomallei (1)<br>Salmonella spp (1)<br>Mycobacterium spp(1)	——	——	AIGA positive	——	None	——	Lymph nodes (1)<br>Blood (1)<br>Muscle (1)	Remission
			41	F(1)	Thailand(1)	Salmonella spp (1)	——	——	AIGA positive	——	None	——	Blood (1)<br>Muscle (1)	Persistent

			51	M(1)	Thailand(1)	VZV(1)<br>Salmonella spp (1)<br>M.aviumcomplex(1)	——	——	AIGA positive	——	None	——	Blood (1)	Persistent
			36	F(1)	Thailand(1)	Cryptococcus neoforman (1)<br>M.abscessus(1)	——	——	AIGA positive	——	None	——	Lymph nodes (1)<br>Blood (1)<br>Lung (1)	Remission
			42	F(1)	Thailand(1)	Salmonella spp (1)<br>M.fortuitum(1)<br>M.abscessus(1)	——	——	AIGA positive	——	None	——	Blood (1)<br>Brain (1)	Remission
			40	M(1)	Thailand(1)	VZV (1)<br>Salmonella spp (1)<br>Burkholderia pseudomallei (1)<br>M.abscessus (1)	——	——	AIGA positive	——	None	——	Blood (1)<br>Spleen (1)	Remission
			64	M(1)	Thailand(1)	Mycobacterium spp (1)	——	——	AIGA positive	——	None	——	Lymph nodes (1)	Persistent
			58	F(1)	Thailand(1)	M.intracellulare (1)<br>M.abscessus (1)	——	——	AIGA positive	——	None	——	Lymph nodes (1)<br>Skin soft tissue (1)	Persistent
			64	M(1)	Thailand(1)	Salmonella spp (1)<br>M.abscessus(1)	——	——	AIGA positive	——	None	——	Lymph nodes (1)<br>Blood (1)	Remission
			43	F(1)	Thailand(1)	M.fortuitum (1)<br>Mycobacterium spp (1)	——	——	AIGA positive	——	None	——	Lymph nodes (1)<br>Blood (1)	Remission
			50	F(1)	Thailand(1)	M.scrofulaceum (1)<br>Mycobacterium spp(1)	——	——	AIGA positive	——	None	——	Bone marrow (1)<br>Blood (1)<br>Skin soft tissue (1)	Remission
			63	F(1)	Thailand(1)	M.abscessus (1)	——	——	AIGA positive	——	None	——	Lymph nodes (1)	Remission
			46	F(1)	Thailand(1)	Salmonella spp (1)<br>Cryptococcus neoformans (1)<br>M.abscessus (1)	——	——	AIGA positive	——	None	——	Joint (1)<br>Blood (1)	Remission
29	Hanitsch LG, 2015[29]	1	65	F(1)	German (1)	M. avium (1)<br>Salmonella spp (1)	Sulfadiazine/pyrimethamine, rifampicin, myambutol and clarithromycin. Deteriorate	——	AIGA positive	Inhibited pSTAT-1	None	——	Lung (1)<br>Pancreas (1)<br>Lymph nodes (1)	Persistent
30	Liu TT, 2016 [30]	1	59	M(1)	China (1)	M. abscessus (1)	Clarithromycin, Doxycyclin, Ceftibuten	——	AIGA positive	——	Rituximab	——	Lung (1)<br>Skin (1)<br>Lymph nodes (1)	Remission
31	Nishimura T, 2015 [31]	1	65	F(1)	Japan (1)	M. avium Complex (1)	Rifampin, ethambutol, clarithromycin, kanamycin, levofloxacin, gatifloxacin	——	AIGA positive	——	None	——	Lung (1)<br>Skin (1)<br>Liver (1)<br>Spleen (1)	Relapse
32	Chi CY, 2016 [32]	45	Mean 59<br>(38-87)	M(21<br>)<br>F(24)	China (45)	M. abscessus (14)<br>M. fortuitum (1)<br>M. chelonae (1)<br>M. avium complex (13)<br>M. kansasii (3)<br>M. scrofulaceum (2)<br>M. gordonae (2)<br>M. terraecomplex (1)<br>Mycobacterium szulgai (1)<br>NTM Species not specified (14)<br>Non-typhoid Salmonella species (18)<br>Legionella pneumophila (1)<br>Staphylococcus aureus(1)<br>Penicillium marneffei(4)<br>Aspergillus species(1)<br>Cryptococcus neoformans (1)<br>Epstein-Barr virus(1)<br>Hepatitis C virus (5)<br>Herpes simplex virus 1(13)<br>Herpes simplex virus 2 (5)<br>Cytomegalovirus(16)<br>Varicella-zoster virus(14)<br>Epstein-Barr virus (18)	Ethambutol, rifampin, rifabutin, tetracyclines, linezolid, ceftibuten	Most cases (95.6%) had an initial nAIGA titer ≥10–4 dilution.	AIGA positive	——	None	——	Lymph node (36)<br>Bone (27)<br>Joint (9)<br>Lung (28)<br>Skin (12)<br>Muscle (2)<br>Spleen (4)<br>Liver (4)<br>Peritoneum (4)<br>Genitourinary system (2)<br>Central nervous system (1)<br>Pericardium (1)	Remission(9)<br>Persistent (27)<br>Lost (9)
33	Lee TL, 2016 [33]	1	55	F(1)	Malaysia	M. abscessus (1)<br>M. tuberculosis (1)	Trimethoprim/sulphamethoxazole, levofloxacin, amikacin, cefoxitin, clarithromycin, rifampicin, isoniazid,	——	AIGA positive	Inhibited pSTAT-1	None	——	Eye (1)<br>Lymph node (1)	Persistent
34	Pruetpongpun N, 2016 [2016]	1	72	M(1)	Thailand(1)	T. marneffei (1)<br>M. abscessusin (1)	Isoniazid, rifampicin, pyrazinamide, ethambutol, amikacin, imipenem/cilastatin, clarithromycin, ciprofloxacin; liposomal amphotericin-B + itraconazole;	1:10 000	AIGA positive	——	Methylprednisolone<br>Rituximab	1:10 000 decreased to 1:5000	Lung (1)<br>Skin (1)<br>Lymph nodes (1)	Remission
35	Valour F, 2016 [35]	1	50	F(1)	Laos(1)	M. fortuitum (1)<br>M. intracellulare (1)<br>VZV (1)	Azithromycin, ciprofloxacin, rifabutin, ethambutol, amikacin, moxifloxacin	——	AIGA positive	——	None	——	Lymph nodes (1)	Relapse

36	Kobayashi T, 2016 [36]	3	67	F(1)	Japan (1)	M. avium (1)	Ethambutol, rifabutin, clarithromycin, ciprofloxacin	——	AIGA positive	——	None	——	Blood (1)<br>Lymph nodes (1)<br>Lung<br>Bone(1)	Death
			66	M(1)	Japan (1)	M. avium (1)	Clarithromycin, rifampin, ethambutol	——	AIGA positive	——	None	——	Lymph nodes<br>Bone(1)	Remission
			71	M(1)	Japan (1)	M. kansasii (1)	Isoniazid, rifampin, ethambutol, pyrazinamide	——	AIGA positive	——	None	——	Muscle (1)<br>Bone(1)	Remission
37	Ikeda H, 2016 [37]	1	68	M(1)	Japan (1)	M. avium (1)	Clarithromycin, rifampicin, ethambutol	OD：0.409, （0.037 in a negative control well）<br>728.13 ELISA Units (EU)	AIGA positive	——	None	Decreased to 109.29 EU	Lung (1)<br>Skin (1)<br>Lymph nodes (1)	Remission
38	van de Vosse E, 2017 [38]	1	38	F(1)	Surinam (1)	Histoplasma capsulatum (1)	Itraconazole	——	AIGA positive	——	None	——	Lymph nodes (1)<br>Throat (1)<br>Eye (1)<br>Spleen (1)<br>Liver (1)<br>Skin (1)<br>Soft tissue (1)	Relapse
39	Chang PH, 2017 [39]	1	73	M(1)	China	M. abscessus (1)	Azithromycin, imipenem, doxycycline, levofloxacin.	——	AIGA positive	——	None	——	Parotid gland (1)<br>Lymph nodes (1)	Remission
40	Chetchotisakd P, 2017[40]	10	34	M(1)	Thailand(1)	cryptococcosis (1)<br>NTM (1)	——	1:1,600,000	AIGA positive	——	None	——	Lung (1)	Unknown
			49	F(1)	Thailand(1)	cryptococcosis (1)<br>NTM (1)	——	1:1,600,000	AIGA positive	——	None	——	Blood (1)<br>Meninges (1)<br>Skin (1)	Unknown
			45	F(1)	Thailand(1)	cryptococcosis (1)<br>NTM (1)	——	1:200,000	AIGA positive	——	None	——	Joint (1)<br>Bone (1)	Unknown
			47	F(1)	Thailand(1)	cryptococcosis (1)<br>NTM (1)	——	1:50,000	AIGA positive	——	None	——	Blood (1)	Unknown
			32	M(1)	Thailand(1)	cryptococcosis (1)<br>NTM (1)	——	1:5,000	AIGA positive	——	None	——	Bone (1)	Unknown
			18	F(1)	Thailand(1)	cryptococcosis (1)<br>NTM (1)	——	1:200,000	AIGA positive	——	None	——	Pleura (1)	Unknown
			55	F(1)	Thailand(1)	cryptococcosis (1)<br>NTM (1)	——	1:10,000	AIGA positive	——	None	——	Lung (1)	Unknown
			82	F(1)	Thailand(1)	cryptococcosis (1)<br>NTM (1)	——	1:10,000	AIGA positive	——	None	——	Parotid gland (1)	Unknown
			34	M(1)	Thailand(1)	cryptococcosis (1)<br>NTM (1)	——	1:10,000	AIGA positive	——	None	——	Bone (1)<br>Joint (1)<br>Lung(1)<br>Skin (1)	Unknown
			43	F(1)	Thailand(1)	cryptococcosis (1)<br>NTM (1)	——	1:10,000	AIGA positive	——	None	——	Joint (1)<br>Bone (1)<br>Meninges (1)<br>Skin (1)	Unknown
41	King YA, 2017 [41]	1	74	F(1)	China (1)	M. kansasii (1)	Clarithromycin, rifampin, ethambutol	——	AIGA positive	——	None	——	Bone (1)<br>Skin (1)<br>Lymph nodes (1)	Remission
42	Asakura T, 2017 [42]	1	66	M(1)	Japan (1)	M. genavense (1)	Clarithromycin, ethambutol, rifampin, amikacin	——	AIGA positive	Inhibited pSTAT-1	None	——	Lymph nodes (1)<br>Lung (1)	Remission
43	Tanimizu M, 2017 [43]	1	72	F(1)	Japan (1)	M. avium (1)	Rifabutin, ethambutol, clarithromycin, streptomycin	51,442 AU	AIGA positive	Inhibited pSTAT-1	None	——	Bone (1)<br>Lung (1)<br>Stomach (1)<br>Peritoneum (1)<br>Lymph nodes (1)	Remission
44	Phoompoung P, 2017 [44]	70	mean 50	M(30)<br>F(40)	Thailand (70)	M.abscessus (51)<br>M. avium complex (8)<br>M.fortuitum (7)<br>M.kansasii (1)<br>M.simiae (1)<br>M.haemophilum (1)<br>M.scrofulaceum (1)<br>Non-typhoid Salmonella species (3)<br>tuberculosis (5)<br>nontyphoidal salmonellosis (17)<br>VZV(4)<br>Melioidosis (2)<br>Fungal infection (4)	——	OD value: 3.64<br>(range 1.32–4.61)	AIGA positive	——	None	——	Lymph nodes (51)<br>Blood or bone marrow (23)<br>Sputum (9)<br>Skin or pus (6)<br>Pleural cavity (1)<br>Joint (1)<br>Lung(1)<br>Bowel (1)<br>Cardiac valve (1)<br>Bone (3)<br>Stool (2)<br>Vascular graft (1)<br>Liver (1)	Unknown
45	Wu UI, 2017 [45]	1	43	M(1)	China (1)	M. avium complex (1)<br>Salmonella spp (1)<br>HIV (1)	Tenofovir, lamivudine, and nevirapine for HIV.<br>Ciprofloxacin for Salmonella spp.	959 mg/ml<br>(reference: 15.78 mg/ml).	AIGA positive	Inhibited pSTAT-1	None	——	Joint (1)<br>Blood (1)<br>Bone (1)	Persistent

46	Suárez I, 2017 [46]	1	54	F(1)	Philippines (1)	M.parascrofulaceum (1)	Amikacin, rifampin, clarithromycin, moxifloxacin, linezolid	——	AIGA positive	Inhibited pSTAT-1	None	——	Soft tissue (1)<br>Bone (1)<br>Skin (1)<br>Lung (1)<br>Breast (1)	Relapse
47	Koizumi Y, 2017 [47]	1	67	F(1)	Japan (1)	M. avium complex (1)	Clarithromycin, rifampicin, ethanbutol, streptomycin, sitafloxacin, amikacin, linezolid, azithromycin, levofloxacin. Persistent	137.3 E.U.	AIGA positive	Inhibited pSTAT-1	Rituximab	Decreased to 51.7 E.U.	Lymph nodes (1)<br>Lung (1)<br>Blood(1)<br>Bone (1)<br>Bone marrow (1)	Remission
48	Furuya H, 2017 [48]	1	55	M(1)	Japan (1)	M. intracellulare (1)	Clarithromycin, ethambutol and rifampicin	——	AIGA positive	Inhibited pSTAT-1	Prednisolone<br>Adalimumab	——	Lymph nodes (1)<br>Lung (1)<br>Blood(1)	Relapse
49	Xu H, 2018 [49]	1	54	M(1)	China (1)	T. marneffei (1)<br>M. abscessus (1)	Fluconazole, amphotericin B, itraconazole; Levofloxacin, clarithromycin	——	AIGA positive	——	None	——	Lymph nodes (1)<br>Lung (1)<br>Skin(1)	Remission
50	Hung TC, 2018 [50]	1	31	F(1)	China (1)	M. kansasii (1)<br>M. abscessus (1)	Isoniazid, rifampin, clarithromycin, ethambutol, amikacin, imipenem/cilastatin, levofloxacin	——	AIGA positive	——	None	——	Lymph nodes (1)<br>Lung (1)<br>Bone(1)<br>Blood (1)	Death
51	Koizumi Y,2019 [51]	1	66	M(1)	Japan (1)	M. avium (1)<br>M. avium complex (1)	Clarithromycin, rifampicin, ethambutol, rifabutin, sitafloxacin, streptomycin	1017 ELISA Units (EU)	AIGA positive	Inhibited pSTAT-1	Rituximab<br>Prednisolone	Decrease to 36 E.U.	Lymph nodes (1)<br>Lung (1)<br>Abdominal (1)	Death
52	Aoki A, 2018 [52]	31	mean 66	F(16)<br>M(15<br>)	Japan (31)	M. avium complex (26)<br>M. gordonae (2)<br>M. kansasii (1)<br>M. mantenii (1)<br>M. colombiense (1)<br>M. genavense (1)<br>M. abscessus complex (3)<br>M. fortuitum (2)<br>Salmonella (1)<br>Cytomegalovirus (1)<br>Varicella zoster (1)	——	——	AIGA positive	Inhibited pSTAT-1	Rituximab<br>Recombinant IFN-γ	——	Lung (19)<br>Bone/joints (19)<br>Lymph nodes (15)<br>Bone marrow/blood (15)<br>Skin (8)<br>Spleen/liver (6)<br>Muscle (4)<br>Urinary organ (2)<br>Genitals (1)<br>Central nervous system (1)	Remission(27)<br>Death (4)
53	Miyashita K, 2018 [53]	1	63	F(1)	Japan (1)	M. avium (1)	Clarithromycin, rifampicin, ethambutol, streptomycin	427 E.U.<br>(control: 22 E.U)	AIGA positive	Inhibited pSTAT-1<br>pSTAT1 index: 113, control: 1042	None	——	Lung (1)<br>Bone (1)<br>Lymph nodes (1)<br>Skin (1)<br>Spleen (1)<br>Muscle (1)<br>Urinary organ (1)	Remission
54	Yamada T, 2018 [54]	1	76	M(1)	Japan (1)	M. intracellulare (1)	Clarithromycin, ethambutol, rifampicin, streptomycin	——	AIGA positive	——	None	——	Lymph nodes (1)	Remission
55	Chetchotisakd P, 2018 [55]	8	52	M(1)	Thailand (1)	M. abscessus (1)<br>Tuberculosis (1)<br>Herpes zoster (1)<br>T. marneffei (1)	Clarithromycin, ofloxacin, cefoxitin, imipenem; Persistent	1:100,000	AIGA positive	——	Cyclophosphamide<br>Methylprednisolone<br>Prednisolone	1 : 50,000	Lymph nodes(1)<br>Lung (1)<br>Spleen (1)	Death
			54	M(1)	Thailand (1)	M. abscessus(1)	Clarithromycin, ciprofloxacin, imipenem; Persistent	1:200,000	AIGA positive	——	Cyclophosphamide<br>Methylprednisolone<br>Prednisolone	1 : 5,000	Lymph nodes (1)<br>Lung (1)	Remission
			57	M(1)	Thailand (1)	M. abscessus(1)<br>M. tuberculosis (1)	Azithromycin, ciprofloxac, imipenem; Persistent	1:200,000	AIGA positive	——	Cyclophosphamide<br>Methylprednisolone<br>Prednisolone	1 : 1,000	Lymph nodes (1)<br>Lung (1)<br>Liver (1)	Remission
			41	M(1)	Thailand (1)	M. abscessus (1)<br>Melioidosis (1)	Clarithromycin, ofloxacin, imipenem; Persistent	1:200,000	AIGA positive	——	Cyclophosphamide<br>Methylprednisolone<br>Prednisolone	1 : 1,000	Lymph nodes (1)<br>Nasal septum (1)	Remission
			54	F(1)	Thailand (1)	M. abscessus (1)	Clarithromycin, ciprofloxacin, imipenem; Persistent	1:100,000	AIGA positive	——	Cyclophosphamide<br>Methylprednisolone<br>Prednisolone	1 : 1,000	Lymph nodes (1)<br>Skin (1)	Remission
			65	M(1)	Thailand (1)	M. abscessus (1)<br>Salmonella spp (1)	Imipenem, clarithromycin, ciprofloxacin; Persistent	1:100,000	AIGA positive	——	Cyclophosphamide<br>Methylprednisolone<br>Prednisolone	1 : 5,000	Lymph nodes (1)<br>Lung (1)<br>Bone (1)	Remission
			34	M(1)	Thailand (1)	M. abscessus(1)<br>Cryptococcosis (1)<br>M. tuberculosis (1)	Clarithromycin, ciprofloxacin, linezolid, imipenem; Persistent	1:200,000	AIGA positive	——	Cyclophosphamide<br>Methylprednisolone<br>Prednisolone	1 : 50,000	Lymph nodes (1)<br>Lung (1)<br>Bone (1)	Relapse
			20	F(1)	Thailand (1)	M. abscessusv(1)<br>Cryptococcosis (1)<br>VZV(1)	Azithromycin, ofloxacin, doxycycline, linezolid, imipenem; Persistent	1:400,000	AIGA positive	——	Cyclophosphamide<br>Methylprednisolone<br>Prednisolone	1 : 50,000	Lymph nodes (1)<br>Skin (1)<br>Lung (1)<br>Liver (1)<br>Spleen (1)<br>Pancreas (1)<br>Bone (1)	Remission

56	Wu UI, 2018 [56]	30	mean 60	F(11)<br>M(19)	China (30)	M. tuberculosis (2)<br>M. avium complex (14)<br>M. kansasii (8)<br>M. abscessus (12)<br>M. chelonae (2)<br>M. gordonae (2)<br>Mix NTM (8)<br>Non-typhoidal salmonellosis (9)<br>Cryptococcosis (1)<br>T. marneffei (1)<br>Aspergillosis (1)<br>VZV (12)	——	——	AIGA positive	Inhibited the production of IFN-γ	None	——	Lymph nodes (21)<br>Bone (17)<br>Skin and soft tissue (12)<br>Lung (16)<br>Blood/bone marrow (5)<br>Central nervous system (2)	Unknown
57	Kashihara E, 2019 [57]	1	33	M(1)	Japan (1)	M. kansasii (1)	Isoniazid, ethambutol, rifampin, pyrazinamide	423.6	AIGA positive	——	None	——	Lymph nodes (1)<br>Lung (1)	Remission
58	Liew WK, 2019 [58]	2	16	F(1)	China (1)	M. abscessus (1)	Clindamycin, cefoxitin, amikacin, linezolid, azithromycin	——	AIGA positive	Inhibited pSTAT-1	None	——	Lymph nodes (1)<br>Skin (1)	Remission
			10	M(1)	Malaysia (1)	M. avium complex (1)	Clarithromycin, rifampicin, ethambutol.	——	AIGA positive	——	None	——	Lymph nodes (1)<br>Skin (1)<br>Bone (1)	Remission
59	Wongkamhla T, 2019	1	52	F(1)	Thailand (1)	T. marneffei (1)<br>M. absessus (1)	Amphotericin B, itraconazole; Imipenem, amikacin, clarithromycin, ciprofloxacin, linezolid	——	AIGA positive	——	None	——	Tonsils (1)<br>Oropharynx and larynx (1)	Relapse
60	Ogawa Y, 2019 [60]	1	80	M(1)	Japan (1)	M. avium complex (1)	——	——	AIGA positive	——	None	——	Lymph nodes (1)<br>Skin (1)<br>Bone (1)	Relapse
61	Qiu Y, 2019 [61]	1	24	M(1)	China (1)	Bacillus cereus (1)	Piperacillin, sulbactam, levofloxacin	——	AIGA positive	——	None	——	Bone (1)	Remission
62	Xu X, 2019 [62]	1	43	F(1)	China (1)	M. avium (1)	Clarithromycin, ethambutol, protionamide, amoxicillin clavulanate potassium, amikacin, linezolid, imipenem, moxifloxacin	——	AIGA positive	——	None	——	Skin (1)<br>Bone (1)	Persistent
63	Angkasekwinai N, 2019 [63]	80	median 49.5	F(41)<br>M(39)	Thailand (80)	M. abscessus (33)<br>M. fortuitum (7)<br>M. avium complex (3)<br>M. scrofulaceum (2)<br>Other NTM spp. (3)<br>Salmonella spp. (18)<br>M. tuberculosis (12)<br>Cryptococcosis (3)<br>T. marneffei (2)<br>Histoplasmosis (1)<br>VZV (6)<br>Burkholderia pseudomallei (1)	Amikacin and/or imipenem and/or cefoxitin) followed by azithromycin or clarithromycin and levofloxacin or moxifloxacin or ciprofloxacin	3.14 (1.57–4.38)<br>3.86 (1.38–4.61)	AIGA positive	——	None	——	Lymph nodes (38)<br>Blood (19)	Remission (11)<br>Persistent (20)<br>Replase (28)<br>Death (4)<br>Lost (17)
64	Yeh YK, 2019 [64]	1	64	M(1)	China (1)	M. avium complex (1)	Clarithromycin, rifabutin, ethambutol, amikacin	OD: 0.85<br>(control 0.07)	AIGA positive	——	None	——	Skin (1)<br>Bone (1)<br>Lung (1)	Remission
65	Yerramilli A, 2019 [65]	1	28	M(1)	India (1)	M. abscessus complex (1)<br>M. abscessus (1)	Isoniazid, rifampicin, pyrazinamide, ethambutol, cefoxitin, tigecycline, amikacin	——	AIGA positive	Inhibited pSTAT-1	None	——	Lymph nodes (1)<br>Lung (1)	Relapse
66	Su SS, 2019 [66]	1	62	F(1)	China (1)	T. marneffei (1)<br>M. avium intracellular (1)<br>M. avium (1)	Amphotericin B, itraconazole; Azithromycin, ethambutol, moxifloxacin, rifabutin	The patient (range: 33.13–47.06 ng/mL)<br>healthy subjects (range: 8.41–20.02 ng/mL)	AIGA positive	——	None	——	Lymph nodes (1)<br>Lung (1)<br>Skin (1)<br>Joint (1)<br>Soft tissue (1)	Persistent
67	Namkoong H, 2018 [67]	1	74	M(1)	Japan (1)	M. avium (1)	Rifampicin, ethambutol, clarithromycin	patient, 50.85 EU;<br>control, 25.49 EU	AIGA positive	Inhibited pSTAT-1	None	——	Lymph nodes (1)<br>Lung (1)<br>Liver (1)<br>Cholecyst (1)	Relapse
68	Tanaka S, 2019 [68]	1	79	F(1)	Japan (1)	M. phlei (1)	Rifampicin, ethambutol , clarithromycin, levofloxacin	——	AIGA positive	——	None	——	Lymph nodes (1)<br>Lung (1)<br>Bone (1)	Remission
69	Rujirachun P, 2020 [69]	1	53	M(1)	Thailand (1)	Cryptococcus (1)<br>VZV(1)<br>M. abscessus (1)	Amphotericin B, fluconazole for cryptococcosis; Amikacin, imipenem, azithromycin, levofloxacin for NTM; acyclovir for VZV.	——	AIGA positive	——	None	——	Lymph nodes (1)<br>Lung (1)<br>Thoracic cavity (1)<br>Cerebrospinal fluid (1)<br>Skin (1)<br>Eye (1)	Remission
70	Yaghnam I, 2020 [70]	1	30	F(1)	America(1)	M. avium complex (1)	——	——	AIGA positive	——	None	——	Lymph nodes (1)<br>Lung (1)<br>Esophagus(1)	Persistent

71	Hong GH, 2020[71]	97	Thailand (mean 50) America (mean 46)	F(68) M(29)	Thailand (n77)<br>Cambodia (1)<br>America(1) Philippines (7)<br>Laos (3)<br>Singapore (1)<br>China (3)<br>Vietnam (3)	M. abscessus (28)<br>M. avium complex (24)<br>M. asiaticum (1)<br>M. chelonae (3)<br>M. fortuitum (4)<br>M. goodie (13)<br>M. malmoense (1)<br>M. massiliense (1)<br>M. scrofulaceum (4)<br>NTM (11)<br>M. tuberculosis (12)<br>Salmonellosis (15)<br>VZV (17)<br>B. cepacia (1)<br>B. pseudomallei (5)<br>A. ustus (1)<br>Coccidioides spp (1)<br>C. neoformans (6)<br>H. capsulatum (10)<br>P. lilacinum (1)<br>T. marneffei (5)<br>HSV (2)	——	——	AIGA positive	——	Cyclophosphamide: 8 cases<br>Rituximab: 10 cases	Decreased	Bone (30)<br>Lymph node (59)<br>Lung (31)<br>Skin (34)<br>Blood (22)<br>Liver/biliary tree (17)<br>Spleen (10)<br>Joints (9)<br>Sinus (8)<br>Central nervous system (6)<br>Muscle (3)<br>Genitourinary system （2）<br>Bone marrow （4）	Death (22)<br>Persistent (7)<br>Relapse (2)<br>Remission (61)<br>Lost (15)
72	Nagamura N, 2021 [72]	1	72	M(1)	Japan (1)	Klebsiella pneumoniae (1)<br>M. avium complex(1)	Carithromycin, rifampicin, ethambutol	49.5 E.U<br>(healthy control of 0.5 E.U)	AIGA positive	Inhibited pSTAT-1<br>pSTAT1 index: 41.8 , control: 365	None	——	Bone (1)<br>Lung (1)<br>Kidney(1)	Death (1)
73	Guo J, 2020 [73]	58	Mean 54<br>(22-77)	F(24) M(34)	China (58)	M. abscessus (2)<br>M. avium complex (1)<br>NTM (4)<br>M. tuberculosis (3)<br>Salmonella spp (4)<br>VZV (8)<br>A. baumannii (1)<br>A. veronii (1)<br>B. cepacia (1)<br>E. cloacae (1)<br>K. pneumoniae (3)<br>Aspergillus spp (1)<br>C. albicans (3)<br>T. marneffei (55)<br>CMV (3)<br>EBV (2)<br>HBV (3)<br>C. sinensis (1)	——	——	AIGA positive	——	None	——	Bone (2)<br>Lymph node (6)<br>Lung (21)<br>Skin (12)<br>Blood (14)<br>Liver/biliary tree (1)<br>Joints (2)<br>Bone marrow （4）<br>Pleural （2）	Death (13)<br>Persistent (19)<br>Remission (12)<br>Lost (14)
74	Ahmad TR, 2020 [74]	1	30	F(1)	America(1)	M. aviumcomplex (1)	Rifabutin, ethambutol, clarithromycin	——	AIGA positive	——	Rituximab<br>Methylprednisolone	——	Eye （1）	Remission
75	Yamaba Y, 2020 [75]	1	68	M(1)	Japan (1)	M. avium complex (1)	Clarithromycin, rifampicin, ethambutol, streptomycin, Sitafloxacin	1,210.97 E.U.<br>(control: 1.91 E.U.)	AIGA positive	Inhibited pSTAT-1<br>pSTAT1 index: 17.76 , control: 556.59	None	——	Bone (1)<br>Lung (1)<br>Liver/biliary tree (1)<br>Spleen (1)<br>Abdominal (1)	Relapse
76	Chaononghin S, 2020 [76]	1	55	M(1)	Thailand (1)	M. scrofulaceum (1)	Amikacin, rifampicin, ethambutol, clarithromycin, trimethoprim/sulfamethoxazole.	1:200,000<br>(reference range <1:100).	AIGA positive	——	Rituximab	——	Bloode (1)<br>Lymph node (1)<br>Lung (1)	Remission
77	Keragala BSDP, 2020	1	24	M(1)	Sri Lanka (1)	M. scrofulaceum (1)	Isoniazid, rifampicin, pyridoxime, ethambutol, amikacin, imipenum, clarithromycin, ciprofloxacin, larithromycin, ciprofloxacin, linezolid	1:30000.	AIGA positive	——	Immunoglobulin	——	Bloode (1)<br>Spleen (1)<br>Intestinal tract(1)	Remission
78	Weng TP, 2020 [78]	1	71	M(1)	China (1)	M. szulgai (1)	Clarithromycin, ethambutol, rifampin	——	AIGA positive	——	None	——	Pleural （1）<br>Lymph node (1)<br>Lung (1)	Remission
79	Liang XN, 2020 [79]	1	69	M(1)	China (1)	Salmonella spp (1)<br>Mycobacterium spp (1)<br>Candida spp (1)<br>Burkholderia cepacia (1)<br>T. marneffei (1)	Moxifloxacin, clarithromycin, sulfamethoxazole, ethambuto	——	AIGA positive	——	None	——	Skin （1）<br>Lymph node (1)<br>Lung (1)	Persistent
			35	F(1)	Thailand (1)	M. avium complex (1)<br>M. fortuitum (1)	——	——	AIGA positive	——	Cyclophosphamide	Decreased	——	Remission
			59	M(1)	Thailand (1)	M. kansasii (1)<br>Salmonella spp (1)<br>HSV (1)<br>Cryptococcus spp (1)	——	——	AIGA positive	——	Cyclophosphamide	Decreased	——	Remission
			56	F(1)	Thailand (1)	NTM (1)	——	——	AIGA positive	——	Cyclophosphamide	Decreased	——	Remission

80	Laisuan W, 2020 [80]	17	59	M(1)	Thailand (1)	M. abscessus (1)<br>HZV (1)	——	——	AIGA positive	——	Cyclophosphamide	Decreased	——	Remission
			40	M(1)	Thailand (1)	M. abscessus (1)	——	——	AIGA positive	——	Cyclophosphamide	Decreased	——	Remission
			47	F(1)	Thailand (1)	M. manteneii (1)<br>Cryptococcus spp. (1)	——	——	AIGA positive	——	Cyclophosphamide	Decreased	——	Remission
			45	F(1)	Thailand (1)	M. parascofula ceum (1)<br>M. abscessus (1)	——	——	AIGA positive	——	Cyclophosphamide	Decreased	——	Remission
			62	M(1)	Thailand (1)	M. abscessus (1)<br>Salmonella spp (1)<br>HSV (1)<br>Cryptococcus spp (1)	——	——	AIGA positive	——	Cyclophosphamide	Decreased	——	Remission
			46	M(1)	Thailand (1)	M. abscessus (1)	——	——	AIGA positive	——	Cyclophosphamide	Decreased	——	Remission
			52	M(1)	Thailand (1)	M. abscessu (1)<br>Cryptococcus spp (1)<br>Cytomegalovirus (1)	——	——	AIGA positive	——	Cyclophosphamide	Decreased	——	Remission
			64	M(1)	Thailand (1)	M. fortuitum (1)<br>Salmonella spp (1)	——	——	AIGA positive	——	Cyclophosphamide	Decreased	——	Remission
			66	F(1)	Thailand (1)	Salmonella spp (1)<br>HSV (1)<br>HZV (1)	——	——	AIGA positive	——	Rituximab	Decreased	——	Remission
			48	F(1)	Thailand (1)	M. avium complex (1)	——	——	AIGA positive	——	Rituximab	Decreased	——	Remission
			44	F(1)	Thailand (1)	M. szulgai (1)<br>M. angelicum (1)	——	——	AIGA positive	——	Rituximab	Decreased	——	Remission
			79	F(1)	Thailand (1)	M. avium (1)<br>M. avium complex (1)	——	——	AIGA positive	——	Rituximab	Decreased	——	Remission
			51	M(1)	Thailand (1)	M. avium complex (1)	——	——	AIGA positive	——	Rituximab	Decreased	——	Remission
			62	M(1)	Thailand (1)	Salmonella spp (1)	——	——	AIGA positive	——	Rituximab	Decreased	——	Remission
81	Roerden M, 2020 [81]	1	44	F(1)	Asian (1)	Cytomegalovirus (1)<br>Salmonella spp (1)<br>M. intracellulare(1)<br>M. abscessusandgoodii(1)	Azithromycin, rifabutin, ethambutol, valganciclovir, cefuroxime	——	AIGA positive	——	Rituximab	Decreased	Skin（1)<br>Lymph node (1)<br>Lung (1)<br>Digestive system (1)	Remission
82	Liang X, 2020 [82]	1	47	F(1)	China (1)	T. marneffei(1)<br>NTM(1)	Amphotericin B and Itraconazole for T. marneffei; Clarithromycin and moxifloxacin	——	AIGA positive	——	None	——	Skin（1)<br>Lymph node (1)<br>Lung (1)<br>Bone (1)	Relapse
83	Loh KM, 2020 [83]	10	—	—	Singapore(1)	NTM (9)<br>Salmonella spp (2)<br>M. tuberculosis (1)	——	——	AIGA positive	——	None	——	——	Unknown
84	Rocco JM, 2021 [84]	1	38	F(1)	Philippines (1)	M. avium complex (1)<br>M. intracellulare (1)	Azithromycin, ethambutol, amikacin,rifampin, linezolid	——	AIGA positive	Inhibited pSTAT-1	Rituximab<br>Bortezomib	Decreased	Skin（1)<br>Lymph node (1)<br>Muscle (1)<br>Bone (1)	Relapse
85	Tham EH, 2016 [85]	3	46	F(1)	Thailand (1)	M. kansasii (1)<br>Aspergillosis (1)	——	——	AIGA positive	Inhibited pSTAT-1	None	——	Bone (1)	Persistent
			45	F(1)	China (1)	M. avium complex (1)<br>M. kansasii(1)<br>M. haemophilum (1)<br>Salmonella spp (1)<br>Cytomegalovirus(1)	——	——	AIGA positive	Inhibited pSTAT-1	None	——	Skin（1)<br>Lung (1)<br>Bone (1)	Relapse
			63	M(1)	Malaysia (1)	M. kansasii (1)<br>Staphylococcus aureus (1)<br>Pseudomonas aeruginosa (1)	——	——	AIGA positive	Inhibited pSTAT-1	None	——	Spleen（1)<br>Lung (1)<br>Bone (1)	Remission
86	Jin W, 2021 [86]	1	68	M(1)	China (1)	T. marneffei (1)<br>M. abscessusin (1)	Voriconazole, Itraconazole; Clarithromycin and moxifloxacin	1:500.	AIGA positive	——	None	——	Lymph node (1)<br>Lung (1)	Remission
87	Ochoa S, 2021 [87]	1	31	F(1)	Philippines (1)	M. avium (1)	zithromycin, ethambutol, tedizolid, moxifloxacin, clofazimi	1521 mg/dL	AIGA positive	Inhibited pSTAT-1	Rituximab<br>Bortezomib	Decrease to 1069 mg/dL	Lung（1)<br>Lymph node (1)<br>Muscle (1)<br>Bone (1)	Remission
88	Mochizuka Y, 2021 [88]	1	78	M(1)	Japan (1)	M. avium (1)	Rifampicin, ethambutol, clarithromycin, streptomycin	427.8 E.U.<br>(control: 26.4 E.U.)	AIGA positive	Inhibited pSTAT-1	None	——	Lung（1)<br>Lymph node (1)<br>Pleural (1)<br>Bone (1)	Remission
89	Uno S, 2021 [89]	1	77	M(1)	Japan (1)	M. avium (1)	larithromycin, rifampicin, ethambutol, and sitafloxacin	——	AIGA positive	Inhibited pSTAT-1	R-CHOP	Decrease	Liver（1)<br>Lymph node (1)<br>Intestinal tract (1)<br>Bone (1)	Remission
90	Harada M, 2021 [90]	1	65	M(1)	Japan (1)	M. avium (1)	Clarithromycin, rifampicin, and ethambutol, azithromycin, streptomycin, moxifloxacin, Rifabutin	63.5 ELISA unit (E.U.) (cutoff: 0.2 E U.)	AIGA positive	Inhibited pSTAT-1	Recombinant IFN-γ	Not decrease	Lymph node (1)<br>Skin (1)<br>Bone (1)	Persistent

91	Thapa P, 2021 [91]	1	63	M(1)	Laos(1)	M. abscessus (1)<br>M. chelonae (1)	Amikacin, imipenem, tigecycline, clofazimine,	——	AIGA positive	——	None	——	Lymph node (1)<br>Skin (1)<br>Bone (1)<br>Lung	Remission
92	Zeng W, 2021 [92]	1	49	M(1)	China (1)	T. marneffei (1)<br>Burkholderia cepacia (1)	Ceftazidime and levofloxacin; Amphotericin B	50,566 ng/mL	AIGA positive	Inhibited pSTAT-1	None	——	Lymph node (1)<br>Skin (1)<br>Bone (1)<br>Lung	Persistent
93	King EM, 2021 [93]	3	44	F(1)	Philippines (1)	M. avium complex (1)	Azithromycin, ethambutol, and rifabutin. Persistent	19071<br>fluorescent intensity	AIGA positive	——	Rituximab	——	Lung (1)<br>Liver (1)<br>Lymph nodes (1)<br>Bone marrow (1)	Remission
			53	F(1)	Cambodia(1)	M. avium complex (1)	Azithromycin, rifampin, amikacin, moxifloxacin; Persistent	19934<br>fluorescent intensity	AIGA positive	——	Rituximab	——	Abdominal (1)<br>Lymph nodes (1)	Remission
			53	M(1)	Philippines (1)	Salmonella spp (1)<br>M. avium complex (1)<br>M. abscessus (1)<br>Massiliense (1)<br>VZV (1)	Azithromycin, rifabutin, ethambutol, moxifloxacin; Persistent	18278<br>fluorescent intensity	AIGA positive	——	Rituximab	——	Lung (1)<br>Lymph nodes (1)<br>Bone (1)<br>Blood (1)	Remission
94	Puel A, 2013 [94]	5	Median 46<br>(25,59)	F(3)<br>M(2)	Thailand (1)<br>Philippines (1)<br>British (2)<br>South African(1)	M. tuberculosis (5)	——	——	AIGA positive	——	None	——	——	Unknown
95	Imoto W, 2020 [95]	1	55	F(1)	Japan (1)	M. tuberculosis (1)<br>M. fortuitum(1)<br>M. abscessus(1)	Azithromycin, imipenem/cilastatin, levofloxacin, minocycline, linezolid, sitafloxacin	——	AIGA positive	——	None	——	Lung (1)<br>Lymph nodes (1)<br>Eye (1)	Death (1)
96	Dahl VN, 2021 [96]	1	71	F(1)	Thailand (1)	M. avium complex (1)<br>Salmonella spp (1)<br>HSV (1)	Rifampicin, ethambutol, clarithromycin, amikacin	——	AIGA positive	Inhibited pSTAT-1	None	——	Lung (1)<br>Lymph nodes (1)<br>Blood (1)<br>intestinal tract(1)	Persistent

Organs involvement	Number	Percentage
Lymph nodes	442	63.14%
Lung	285	40.71%
Bone	231	33.00%
Skin	197	28.14%
Bloodstream	131	18.71%
Liver	69	9.86%
Bone marrow	58	8.29%
Spleen	47	6.71%
Joint	35	5.00%
Pleural cavity	33	4.71%
Central nervous system	32	4.57%
Muscle	18	2.57%
Pericardium	13	1.86%
Abdominal cavity	12	1.71%
Urogenital system	12	1.71%
Gastrointestinal tract	10	1.43%
Sinus	10	1.43%
Soft tissue	9	1.29%
Eyes	8	1.14%
Pharynx	4	0.57%
Tonsils	3	0.43%
Lacrimal gland	2	0.29%
Pancreas	2	0.29%
Parotid gland	2	0.29%
Appendix	1	0.14%
Breast	1	0.14%
Cholecyst	1	0.14%
Cardiac valve	1	0.14%
