# Supplementary material for: Pathogen spectrum and immunotherapy in patients with anti-IFN-γ autoantibodies: A multicenter retrospective study and systematic review
Source: Front Immunol. 2022 Dec 8;13:1051673. doi: 10.3389/fimmu.2022.1051673 (PMC9772057; doi:10.3389/fimmu.2022.1051673)
Supplement: Supplementary file 2 [file DataSheet_2.pdf]

Supplementary Material

Pathogen spectrum and immunotherapy in patients with anti-IFN-γ autoantibodies: a multicenter retrospective study and systematic review

A total of 1540 articles published in English between January 1, 1985 and September 31, 2021, were identified by systematic literature review. After reviewing the article abstracts, 1399 articles not relevant to AIGAs were excluded. Among the remaining 144 articles, 96 (1-96) were used as data sources.

References

1. Döffinger R, Helbert MR, Barcenas-Morales G, Yang K, Dupuis S, Ceron-Gutierrez L, et al. Autoantibodies to interferon-gamma in a patient with selective susceptibility to mycobacterial infection and organ-specific autoimmunity. Clin Infect Dis. 2004;38(1): e10-4.
2. Höflich C, Sabat R, Rosseau S, Temmesfeld B, Slevogt H, Döcke WD, et al. Naturally occurring anti-IFN-gamma autoantibody and severe infections with *Mycobacterium cheloneae* and *Burkholderia cocovenenans*. Blood. 2004;103(2):673-675.
3. Kampmann B, Hemingway C, Stephens A, Davidson R, Goodsall A, Anderson S, et al. Acquired predisposition to mycobacterial disease due to autoantibodies to IFN-gamma. J Clin Invest. 2005;115(9):2480-8.
4. Patel SY, Ding L, Brown MR, Lantz L, Gay T, Cohen S, et al. Anti-IFN-gamma autoantibodies in disseminated nontuberculous mycobacterial infections. J Immunol. 2005;175(7):4769-76.
5. Tanaka Y, Hori T, Ito K, Fujita T, Ishikawa T, Uchiyama T. Disseminated *Mycobacterium avium complex* infection in a patient with autoantibody to interferon-gamma. Intern Med. 2007;46(13):1005-9.
6. Koya T, Tsubata C, Kagamu H, Koyama K, Hayashi M, Kuwabara K, et al. Anti-interferon-gamma autoantibody in a patient with disseminated *Mycobacterium avium complex*. J Infect Chemother. 2009;15(2):118-22.
7. Baerlecken N, Jacobs R, Stoll M, Schmidt RE, Witte T. Recurrent, multifocal *Mycobacterium avium-intercellulare* infection in a patient with interferon-gamma autoantibody. Clin Infect Dis. 2009 Oct 1;49(7):e76-8.
8. Tang BS, Chan JF, Chen M, Tsang OT, Mok MY, Lai RW, et al. Disseminated penicilliosis, recurrent bacteremic nontyphoidal salmonellosis, and burkholderiosis associated with acquired immunodeficiency due to autoantibody against gamma interferon. Clin Vaccine Immunol. 2010;17(7):1132-8.
9. Kampitak T, Suwanpimolkul G, Browne S, Suankratay C. Anti-interferon-γ autoantibody and opportunistic infections: case series and review of the literature. Infection. 2011;39(1):65-71.
10. Browne SK, Burbelo PD, Chetchotisakd P, Suputtamongkol Y, Kiertiburanakul S, Shaw PA, et al. Adult-onset immunodeficiency in Thailand and Taiwan. N Engl J Med. 2012;367(8):725-34.
11. Browne SK, Zaman R, Sampaio EP, Jutivorakool K, Rosen LB, Ding L, et al. Anti-CD20 (rituximab) therapy for anti-IFN-γ autoantibody-associated nontuberculous mycobacterial infection. Blood. 2012;119(17):3933-9.
12. Picque JB, Blot M, Binois R, Jeudy G, Simonet AL, Cagnon J, et al. Recurrent atypical mycobacterial infections in the adult: think of autoantibodies against interferon-gamma. Rev Med Interne. 2012;33(2):103-6.
13. Tanaka H, Yamaguchi E, Fukuoka T, Ohbayashi Y, Sato M, Yokoi T. A case of disseminated

nontuberculous mycobacteriosis and cerebellar toxoplasmosis with autoantibody to interferon-γ. Sarcoidosis Vasc Diffuse Lung Dis. 2013;30(4):312-6.

14. Nei T, Okabe M, Mikami I, Koizumi Y, Mase H, Matsuda K, et al. A non-HIV case with disseminated *Mycobacterium kansasii* disease associated with strong neutralizing autoantibody to interferon-γ. Respir Med Case Rep. 2012;8: 10-3.
15. Chi CY, Chu CC, Liu JP, Lin CH, Ho MW, Lo WJ, et al. Anti-IFN-γ autoantibodies in adults with disseminated nontuberculous mycobacterial infections are associated with HLA-DRB1*16:02 and HLA-DQB1*05:02 and the reactivation of latent varicella-zoster virus infection. Blood. 2013;121(8):1357-66.
16. Wongkulab P, Wipasa J, Chaiwarith R, Supparatpinyo K. Autoantibody to interferon-gamma associated with adult-onset immunodeficiency in non-HIV individuals in Northern Thailand. PLoS One. 2013;8(9): e76371.
17. Ishii T, Tamura A, Matsui H, Nagai H, Akagawa S, Hebisawa A, et al. Disseminated *Mycobacterium avium complex* infection in a patient carrying autoantibody to interferon-γ. J Infect Chemother. 2013;19(6):1152-7.
18. Poulin S, Corbeil C, Nguyen M, St-Denis A, Côté L, Le Deist F, et al. Fatal *Mycobacterium colombiense*/*cytomegalovirus* coinfection associated with acquired immunodeficiency due to autoantibodies against interferon gamma: a case report. BMC Infect Dis. 2013; 13:24.
19. Chan JF, Trendell-Smith NJ, Chan JC, Hung IF, Tang BS, Cheng VC, et al. Reactive and infective dermatoses associated with adult-onset immunodeficiency due to anti-interferon-gamma autoantibody: Sweet's syndrome and beyond. Dermatology. 2013;226(2):157-66.
20. Chan JF, Yee KS, Tang BS, Cheng VC, Hung IF, Yuen KY. Adult-onset immunodeficiency due to anti-interferon-gamma autoantibody in mainland Chinese. Chin Med J (Engl). 2014;127(6):1189-90.
21. Chetchotisakd P, Anunnatsiri S. Linezolid in the treatment of disseminated nontuberculous mycobacterial infection in anti-interferon-gamma autoantibody-positive patients. Southeast Asian J Trop Med Public Health. 2014;45(5):1125-31.
22. DeLeon TT, Chung HH, Opal SM, Dworkin JD. *Mycobacterium avium complex* empyema in a patient with interferon gamma autoantibodies. Hawaii J Med Public Health. 2014;73(9 Suppl 1):15-7.
23. Suzuki K, Terada J, Sasaki Y, Kawasaki T, Naito Y, Sakurai T, et al. Pulmonary *Mycobacterium fortuitum* infection with cervical lymphadenitis in a patient carrying autoantibodies to interferon-γ. Intern Med. 2014;53(12):1361-4.
24. Czaja CA, Merkel PA, Chan ED, Lenz LL, Wolf ML, Alam R, et al. Rituximab as successful adjunct treatment in a patient with disseminated nontuberculous mycobacterial infection due to acquired anti-interferon-γ autoantibody. Clin Infect Dis. 2014;58(6):e115-8.
25. O'Connell E, Rosen LB, LaRue RW, Fabre V, Melia MT, Auwaerter PG, et al. The first US domestic report of disseminated Mycobacterium avium complex and anti-interferon-γ autoantibodies. J Clin Immunol. 2014;34(8):928-32.
26. Hase I, Morimoto K, Sakagami T, Kazumi Y, Ishii Y, van Ingen J. Disseminated *Mycobacterium gordonae* and *Mycobacterium mantenii* infection with elevated anti-IFN-γ neutralizing autoantibodies. J Infect Chemother. 2015;21(6):468-72.
27. Otome O, O'Reilly M, Lim L. Disseminated *Mycobacterium haemophilum* skeletal disease in a patient with interferon-gamma deficiency. Intern Med J. 2015;45(10):1073-6.
28. Pithukpakorn M, Roothumnong E, Angkasekwinai N, Suktitipat B, Assawamakin A, Luangwedchakarn V, et al. HLA-DRB1 and HLA-DQB1 Are Associated with Adult-Onset Immunodeficiency with Acquired Anti-Interferon-Gamma Autoantibodies. PLoS One.

2015;10(5): e0128481.
29. Hanitsch LG, Löbel M, Müller-Redetzky H, Schürmann M, Suttorp N, Unterwalder N, et al. Late-Onset Disseminated *Mycobacterium avium intracellulare complex* Infection (MAC), Cerebral Toxoplasmosis and Salmonella Sepsis in a German Caucasian Patient with Unusual Anti-Interferon-Gamma IgG1 Autoantibodies. J Clin Immunol. 2015;35(4):361-5.
30. Liu TT, Weng SW, Wang MC, Huang WT. Nontuberculous mycobacterial infection with concurrent IgG4-related lymphadenopathy. APMIS. 2016;124(3):216-20.
31. Nishimura T, Fujita-Suzuki Y, Yonemaru M, Ohkusu K, Sakagami T, Carpenter SM, et al. Recurrence of disseminated *Mycobacterium avium complex* disease in a patient with anti-gamma interferon autoantibodies by reinfection. J Clin Microbiol. 2015;53(4):1436-8.
32. Chi CY, Lin CH, Ho MW, Ding JY, Huang WC, Shih HP, et al. Clinical manifestations, course, and outcome of patients with neutralizing anti-interferon-γ autoantibodies and disseminated nontuberculous mycobacterial infections. Medicine (Baltimore). 2016;95(25):e3927.
33. Lee TL, Agrawal R, Tan JY, Ong KH, Wong CS, Ho SL. Disseminated nontuberculous mycobacterial infection with multifocal retinitis and vasculitis in an immunocompromised patient with anti-IFN-ɣ autoantibodies. J Ophthalmic Inflamm Infect. 2016;6(1):39.
34. Pruetpongpun N, Khawcharoenporn T, Damronglerd P, Suthiwartnarueput W, Apisarnthanarak A, Rujanavej S, et al. Disseminated *Talaromyces marneffei* and *Mycobacterium abscessus* in a Patient with Anti-Interferon-γ Autoantibodies. Open Forum Infect Dis. 2016 May 10;3(2): ofw093.
35. Valour F, Perpoint T, Sénéchal A, Kong XF, Bustamante J, Ferry T, et al. Interferon-γ Autoantibodies as Predisposing Factor for Nontuberculous Mycobacterial Infection. Emerg Infect Dis. 2016;22(6):1124-1126.
36. Kobayashi T, Morino E, Takasaki J, Nagahara Y, Sugiyama H. Nontuberculous Mycobacterial Osteomyelitis in Human Immunodeficiency Virus-Negative Patients: A Case Series. Jpn J Infect Dis. 2016;69(2):149-50.
37. Ikeda H, Nakamura K, Ikenori M, Saito T, Nagamine K, Inoue M, et al. Severe Disseminated Mycobacterium avium Infection in a Patient with a Positive Serum Autoantibody to Interferon-γ. Intern Med. 2016;55(20):3053-3058.
38. van de Vosse E, van Wengen A, van der Meide WF, Visser LG, van Dissel JT. A 38-year-old woman with necrotising cervical lymphadenitis due to Histoplasma capsulatum. Infection. 2017;45(6):917-920.
39. Chang PH, Chuang YC. Anti-interferon-γ autoantibody-associated disseminated *Mycobacterium abscessus* infection mimicking parotid cancer with multiple metastases: A case report. Medicine (Baltimore). 2017;96(39):e8118.
40. Chetchotisakd P, Anunnatsiri S, Nithichanon A, Lertmemongkolchai G. Cryptococcosis in Anti-Interferon-Gamma Autoantibody-Positive Patients: a Different Clinical Manifestation from HIV-Infected Patients. Jpn J Infect Dis. 2017;70(1):69-74.
41. King YA, Hu CH, Lee YJ, Lin CF, Liu D, Wang KH. Disseminated cutaneous Mycobacterium kansasii infection presenting with Rosai-Dorfman disease-like histological features in a patient carrying anti-interferon-γ autoantibodies. J Dermatol. 2017;44(12):1396-1400.
42. Asakura T, Namkoong H, Sakagami T, Hasegawa N, Ohkusu K, Nakamura A. Disseminated Mycobacterium genavense Infection in Patient with Adult-Onset Immunodeficiency. Emerg Infect Dis. 2017;23(7):1208-1210.
43. Tanimizu M, Mizuno K, Hashimoto M. Disseminated Nontuberculous Mycobacterial Infection in a Patient with Anti-IFN-γ Autoantibodies. Acta Med Okayama. 2017;71(6):547-552.
44. Phoompoung P, Ankasekwinai N, Pithukpakorn M, Foongladda S, Umrod P, Suktitipat B, et al. Factors associated with acquired Anti IFN- γ autoantibody in patients with nontuberculous

mycobacterial infection. PLoS One. 2017;12(4): e0176342.
45. Wu UI, Hung CC, Chang SY, Jhong YT, Sun HY, Wang JT, et al. Neutralizing antiinterferon-γ autoantibodies causing disseminated Mycobacterium avium complex infection in an HIV-infected patient on successful combination antiretroviral therapy. AIDS. 2017;31(18):2557-2559.
46. Suárez I, Lehmann C, Gruell H, Graeb J, Kochanek M, Fätkenheuer G, et al. Repurposing QuantiFERON for Detection of Neutralizing Interferon-γ Autoantibodies in Patients with Nontuberculous Mycobacterial Infections. Clin Infect Dis. 2017;65(3):518-521.
47. Koizumi Y, Sakagami T, Nishiyama N, Hirai J, Hayashi Y, Asai N, et al. Rituximab Restores IFN-γ-STAT1 Function and Ameliorates Disseminated Mycobacterium avium Infection in a Patient with Anti-Interferon-γ Autoantibody. J Clin Immunol. 2017;37(7):644-649.
48. Furuya H, Ikeda K, Miyachi K, Nakamura K, Suzuki K, Furuta S, et al. SAPHO syndrome-like presentation of disseminated nontuberculous mycobacterial infection in a case with neutralizing anti-IFNγ autoantibody. Rheumatology (Oxford). 2017;56(7):1241-1243.
49. Xu H, Liu D, He X, Zheng D, Deng Y. Sweet's Syndrome Associated with *Talaromyces marneffei* and *Mycobacterium abscessus* Infection Due to Anti-interferon-gamma Autoantibodies. Indian J Dermatol. 2018;63(5):428-430.
50. Hung TC, Chen SC, Liao KS, Cheng SH, Chang SL. Anti-interferon-γ auto-anti-body and disseminated non-tuberculous mycobacteria infections in thyroid cancer: a case report. QJM. 2018;111(9):647-648.
51. Koizumi Y, Sakagami T, Minamiguchi H, Makino A, Aoki A, Hodohara K, et al. Chylous ascites, anti-interferon-gamma autoantibody, and angioimmunoblastic T-cell lymphoma: a rare but intriguing connection over Mycobacterium avium. Med Microbiol Immunol. 2019;208(1):33-37.
52. Aoki A, Sakagami T, Yoshizawa K, Shima K, Toyama M, Tanabe Y, et al. Clinical Significance of Interferon-γ Neutralizing Autoantibodies Against Disseminated Nontuberculous Mycobacterial Disease. Clin Infect Dis. 2018;66(8):1239-1245.
53. Miyashita K, Matsuura S, Tajima K, Tajima S, Aoki A, Sakagami T, et al. Disseminated *Mycobacterium avium* Infection Presenting with Bladder Lesions in a Patient with Interferon-γ-neutralizing Autoantibodies. Intern Med. 2018;57(20):3041-3045.
54. Yamada T, Iwakami SI, Hara SAM, Iwakami N, Nakamura A, Suzuki Y, et al. Disseminated non-tuberculous mycobacterial infection caused by anti-interferon-γ autoantibodies in a patient of very advanced age. Geriatr Gerontol Int. 2018;18(7):1132-1133.
55. Chetchotisakd P, Anunnatsiri S, Nanagara R, Nithichanon A, Lertmemongkolchai G. Intravenous Cyclophosphamide Therapy for Anti-IFN-Gamma Autoantibody-Associated Mycobacterium abscessus Infection. J Immunol Res. 2018; 2018: 6473629.
56. Wu UI, Chuang YC, Sheng WH, Sun HY, Jhong YT, Wang JY, et al. Use of QuantiFERON-TB Gold In-tube assay in screening for neutralizing anti-interferon-γ autoantibodies in patients with disseminated nontuberculous mycobacterial infection. Clin Microbiol Infect. 2018;24(2):159-165.
57. Kashihara E, Fujita K, Uchida N, Yamamoto Y, Mio T, Koyama H. Case Report: Disseminated *Mycobacterium kansasii* Disease in a Patient with Anti-Interferon-Gamma Antibody. Am J Trop Med Hyg. 2019;101(5):1066-1069.
58. Liew WK, Thoon KC, Chong CY, Tan NWH, Cheng DT, Chan BSW, et al. Juvenile-Onset Immunodeficiency Secondary to Anti-Interferon-Gamma Autoantibodies. J Clin Immunol. 2019;39(5):512-518.
59. Wongkamhla T, Chongtrakool P, Jitmuang A. A case report of *Talaromyces marneffei* Oro-pharyngo-laryngitis: a rare manifestation of Talaromycosis. BMC Infect Dis. 2019;19(1):1034.
60. Ogawa Y, Hasebe R, Ohnuma T, Sano S, Mitsui H, Shimada S, et al. Acute generalized

exanthematous pustulosis associated with anti-interferon-γ neutralizing autoantibody-positive disseminated nontuberculous mycobacterial infection. Eur J Dermatol. 2019;29(3):339-341.
61. Qiu Y, Zhang J, Li B, Shu H. Bacillus cereus isolated from a positive bone tissue culture in a patient with osteolysis and high-titer anti-interferon-γ autoantibodies: A case report. Medicine (Baltimore). 2019;98(43):e17609.
62. Xu X, Lao X, Zhang C, Cao C, Ding H, Pang Y, et al. Chronic *Mycobacterium avium* skin and soft tissue infection complicated with scalp osteomyelitis possibly secondary to anti-interferon-γ autoantibody formation. BMC Infect Dis. 2019;19(1):203.
63. Angkasekwinai N, Suputtamongkol Y, Phoompoung P, Pithukpakorn M, Wongswat E, Umrod P, et al. Clinical outcome and laboratory markers for predicting disease activity in patients with disseminated opportunistic infections associated with anti-interferon-γ autoantibodies. PLoS One. 2019;14(4): e0215581.
64. Yeh YK, Ding JY, Ku CL, Chen WC. Disseminated *Mycobacterium avium* complex infection mimicking malignancy in a patient with anti-IFN-γ autoantibodies: a case report. BMC Infect Dis. 2019;19(1):909.
65. Yerramilli A, Huang GKL, Griffin DWJ, Kong KL, Muhi S, Muttucumaru RS, et al. Disseminated Nontuberculous Mycobacterial Infection Associated with Acquired Immunodeficiency Due to Anti-Interferon γ Autoantibodies. Open Forum Infect Dis. 2019;6(4): ofz131.
66. Su SS, Zhang SN, Ye JR, Xu LN, Lin PC, Xu HY, et al. Disseminated *Talaromyces marneffei* and *Mycobacterium avium* Infection Accompanied Sweet's Syndrome in A Patient with Anti-Interferon-γ Autoantibodies: A Case Report. Infect Drug Resist. 2019;12: 3189-3195.
67. Namkoong H, Asakura T, Ishii M, Yoda S, Masaki K, Sakagami T, et al. First report of hepatobiliary *Mycobacterium avium* infection developing obstructive jaundice in a patient with neutralizing anti-interferon-gamma autoantibodies. New Microbes New Infect. 2018; 27:4-6.
68. Tanaka S, Hoshino Y, Sakagami T, Fukano H, Matsui Y, Hiranuma O. Pathogenicity of *Mycolicibacterium phlei*, a non-pathogenic nontuberculous mycobacterium in an immunocompetent host carrying anti-interferon gamma autoantibodies: a case report. BMC Infect Dis. 2019;19(1):454.
69. Rujirachun P, Sangwongwanich J, Chayakulkeeree M. Triple infection with Cryptococcus, varicella-zoster virus, and *Mycobacterium abscessus* in a patient with anti-interferon-gamma autoantibodies: a case report. BMC Infect Dis. 2020; 20(1):232.
70. Yaghnam I, Jain R, Golamari R, Clarke K. Acquired tracheoesophageal fistula in disseminated *Mycobacterium avium complex* associated with anti-interferon-gamma autoantibodies. BMJ Case Rep. 2020;13(11): e235661.
71. Hong GH, Ortega-Villa AM, Hunsberger S, Chetchotisakd P, Anunnatsiri S, Mootsikapun P, et al. Natural History and Evolution of Anti-Interferon-γ Autoantibody-Associated Immunodeficiency Syndrome in Thailand and the United States. Clin Infect Dis. 2020;71(1):53-62.
72. Nagamura N, Imada T. Anti-interferon-gamma autoantibody related disseminated nontuberculous mycobacteriosis with pathological features of immunoglobulin G4-related disease. Immunol Med. 2021:1-6.
73. Guo J, Ning XQ, Ding JY, Zheng YQ, Shi NN, Wu FY, et al. Anti-IFN-γ autoantibodies underlie disseminated *Talaromyces marneffei* infections. J Exp Med. 2020;217(12):e20190502.
74. Ahmad TR, Ashraf DC, Seitzman GD. Corneal Deposits in a Patient with Anti-Interferon-γ Autoantibody Syndrome. JAMA Ophthalmol. 2020;138(3):310-311.
75. Yamaba Y, Takakuwa O, Wang Z, Saito M, Kawae D, Yoshihara M, et al. Disseminated *Mycobacterium avium* Infection Complicated with Chylous Ascites in a Patient with Neutralizing

Autoantibodies to Interferon-γ. Intern Med. 2020;59(24):3195-3200.
76. Chaononghin S, Visuttichaikit S, Apisarnthanarak A, Khawcharoenporn T. Disseminated *Mycobacterium scrofulaceum* Infection in a Patient with Anti-Interferon-γ Autoantibodies: A Case Report and Review of the Literature. Int J Mycobacteriol. 2020;9(1):91-94.
77. Keragala BSDP, Gunasekera CN, Yesudian PD, Guruge C, Dissanayaka BS, Liyanagama DP, et al. Disseminated Mycobacterium simiae infection in a patient with adult-onset immunodeficiency due to anti-interferon-gamma antibodies - a case report. BMC Infect Dis. 2020;20(1):258.
78. Weng TP, Syue LS, Lee NY. Disseminated Mycobacterium szulgai infection in a patient with anti-interferon-gamma autoantibodies. IDCases. 2020;21:e00848.
79. Liang XN, Bin YF, Lai GT, Li YH, Zhang JQ, Zhong XN, et al. Non-tuberculous mycobacterial infection and reactive dermatosis associated with adult-onset immunodeficiency due to anti-interferon-gamma autoantibodies: A case report. Medicine (Baltimore). 2020;99(36):e21738.
80. Laisuan W, Pisitkun P, Ngamjanyaporn P, Suangtamai T, Rotjanapan P. Prospective Pilot Study of Cyclophosphamide as an Adjunct Treatment in Patients with Adult-Onset Immunodeficiency Associated With Anti-interferon-γ Autoantibodies. Open Forum Infect Dis. 2020;7(2): ofaa035.
81. Roerden M, Döffinger R, Barcenas-Morales G, Forchhammer S, Döbele S, Berg CP. Simultaneous disseminated infections with intracellular pathogens: an intriguing case report of adult-onset immunodeficiency with anti-interferon-gamma autoantibodies. BMC Infect Dis. 2020;20(1):828.
82. Liang X, Si L, Li Y, Zhang J, Deng J, Bai J, et al. *Talaromyces marneffei* infection relapse presenting as osteolytic destruction followed by suspected nontuberculous mycobacterium infection during 6 years of follow-up: A case update. Int J Infect Dis. 2020; 93:208-210.
83. Loh KM, Zhong Y, Lin L, Chan M, Cherng BPZ, Yew HS, et al. When to Test for Anti-Interferon-γ Autoantibody? Clin Infect Dis. 2020;71(7): e199.
84. Rocco JM, Rosen LB, Hong GH, Treat J, Kreuzburg S, Holland SM, et al. Bortezomib treatment for refractory nontuberculous mycobacterial infection in the setting of interferon gamma autoantibodies. J Transl Autoimmun. 2021; 4:100102.
85. Tham EH, Huang CH, Soh JY, Thayalasingam M, Lee AJ, Lum LH, et al. Neutralizing Anti-Interferon-Gamma Autoantibody Levels May Not Correlate With Clinical Course of Disease. Clin Infect Dis. 2016;63(4):572-3.
86. Jin W, Liu J, Chen K, Shen L, Zhou Y, Wang L. Coinfection by *Talaromyces marneffei* and *Mycobacterium abscessus* in a human immunodeficiency virus-negative patient with anti-interferon-γ autoantibody: a case report. J Int Med Res. 2021;49(1):300060520976471.
87. Ochoa S, Ding L, Kreuzburg S, Treat J, Holland SM, Zerbe CS. Daratumumab (Anti-CD38) for Treatment of Disseminated Nontuberculous Mycobacteria in a Patient with Anti-Interferon-γ Autoantibodies. Clin Infect Dis. 2021;72(12):2206-2208.
88. Mochizuka Y, Kono M, Hirama R, Oshima Y, Takeda K, Tsutsumi A, et al. Endobronchial Lesions from Disseminated Mycobacterium avium Infection in a Patient with Anti-interferon-gamma Autoantibodies. Intern Med. 2021;60(20):3267-3272.
89. Uno S, Uehara E, Kimura T, Sakagami T, Namkoong H, Uchida S, et al. R-CHOP Chemotherapy for Disseminated *Mycobacterium avium Complex* Disease due to Anti-Interferon-Gamma Autoantibodies: A Case Report. Open Forum Infect Dis. 2021;8(6): ofab181.
90. Harada M, Furuhashi K, Karayama M, Suzuki Y, Hozumi H, Enomoto N, et al. Subcutaneous injection of interferon gamma therapy could be useful for anti-IFN-γ autoantibody associated disseminated nontuberculous mycobacterial infection. J Infect Chemother. 2021;27(2):373-378.
91. Thapa P, Mohr K, Campbell KK, Saccente M. Sweet Syndrome precipitated by Mycobacterium abscessus in a Laotian Man with Autoantibodies to Interferon Gamma. J Investig Med High

Impact Case Rep. 2021; 9:2324709621990771.
92. Zeng W, Qiu Y, Tang M, Zhang H, Pan M, Tang S, et al. *Talaromyces marneffei* and *Burkholderia cepacia* Co-Infection in a HIV-Uninfected Patient with Anti-Interferon-γ Autoantibodies. Infect Drug Resist. 2021; 14:2173-2177.
93. King EM, Weaver VK, Kestler MH. Treatment Dilemmas in Disseminated Nontuberculous Mycobacterial Infections with Interferon-gamma Autoantibodies. Open Forum Infect Dis. 2021;8(7): ofab253.
94. Puel A, Casanova JL. Autoantibodies against cytokines: back to human genetics. Blood. 2013;121(8):1246-7.
95. Imoto W, Yamada K, Hajika Y, Okamoto K, Myodo Y, Niki M, et al. Disseminated Mycobacterium abscessus subsp. massiliense infection in a Good's syndrome patient negative for human immunodeficiency virus and anti-interferon-γ autoantibody: a case report. BMC Infect Dis. 2020;20(1):431.
96. Dahl VN, Nielsen BU, Wejse CM, Marquart HV, Bay JT, von Stemann JH, et al. Disseminated Mycobacterium avium complex infection in a woman with anti-interferon- γ autoantibodies. IDCases. 2021;26:e01300.

Supplementary Table 1. Clinical characteristics and outcome of the 79 participants in the retrospective study

**Variable**	**n=79**
Age (year)	49 (45, 60)
Sex, female n (%)	36 (45.6)
AIGAs titers (ng/mL)	49327.9 (25222.9, 84167.8)
No. of infecting pathogens*	2 (1, 7)
No. of involved sites	3 (2, 6)
WBC×10^9 cells/L	17.9 (13.8, 22.6)
N×10^9 cells/L	12.3 (8.53, 15.2)
L×10^9 cells/L	2.5 (1.6, 3.6)
HGB g/L	91.0 (78.4, 104.5)
ESR mm/h	104.0 (96.2, 119.8)
CRP mg/L	152.9 (126.3, 200.0)
$CD4^+$ T-cell cells/μL	660 (430, 999)
$CD8^+$ T-cell cells/μL	489 (321, 902)
$CD3^+$ T-cell cells/μL	897 (586, 1591)
IgG g/L	22.7 (19.3, 29.7)
IgA g/L	2.7 (1.8, 4.0)
IgM g/L	1.3 (0.97, 2.0)
Globulin g/L	45.9 (36.8, 53.55)
Prognosis and outcomes n (%)	

Cured	17 (21.5%)
Persistent infection	23 (29.1%)
Relapse infection	27 (34.2%)
Death	12 (15.2%)

Data are expressed as median ± interquartile range.

Abbreviations: AIGAs, Anti-IFN-γ auto-antibodies, WBC, White blood cell; N, neutrophil counts; L, Lymphocyte counts; HGB, hemoglobin; ESR, erythrocyte sedimentation rate; CRP, C-reactive protein; Ig, immunoglobulin.

Normal range: IgG: 8−18 g/L, IgA: 2.01−2.69 g/L, IgM: 0.84–1.32 g/L, CD4+ T-cell: 410−1590 cells/μL, CD8+ T-cell: 190−1140 cells/μL, CD3+ T-cell: 690−2540 cells/μL.
