# Supplementary material for: Pathogen spectrum and immunotherapy in patients with anti-IFN-γ autoantibodies: A multicenter retrospective study and systematic review
Source: Front Immunol. 2022 Dec 8;13:1051673. doi: 10.3389/fimmu.2022.1051673 (PMC9772057; doi:10.3389/fimmu.2022.1051673)
Supplement: Supplementary file 3 [file Table_2.docx]

**Supplementary Table 2.** Summary of treatment and outcomes of 62 Patients with anti-interferon-γ autoantibodies

| **Patient ID [reference]** | **Sex, age (years)** | **Infecting pathogens** | **Antimicrobial treatment** | **Antimicrobial treatment outcome** | **AIGA treatment** | **AIGA titer change** | **Toxic side effects** | **Follow-up (months)** | **Outcome** |
| --- | --- | --- | --- | --- | --- | --- | --- | --- | --- |
| P1 ^[8]^ | M, 47 | *M. tuberculosis*, NTM | RFP, INH, PZA, EMB, for TB for 12 months;  INH, PZA, EMB, AMK, DOX, CLR for NTM for 3 years | Persistent | IFN-γ: 90 mg, 3 times/week for 3 years | No decrease | ··· | 36 | Death |
| P2 ^[9]^ | F, 46 | MAI | RFP, CLR, EMB, CFZ for 3 years | Deteriorated | IFN-γ: 100 µg/m^2^, 3 times/week for 42 months | No decrease | ··· | 42 | Remission |
| P3 ^[10]^ | M, 65 | *M. avium* | CLR, RFP, EMB, AZM, SM, MFLX | Persistent | IFN-γ: 500,000 IU | No decrease | ··· | ··· | Remission |
| P4 ^[11]^ | F, 46 | *M. abscessus*  MAC | CLR, EMB, INH, LZD, MXFX, TGC for 5 years | Persistent | IFN-γ | ··· | ··· | 1 | Remission |
|  |  |  |  |  | RTX: 375 mg/m^2^/week, 7 doses | 80% decrease | ··· | 2 |  |
| P5 ^[11]^ | F, 69 | *M. abscessus* | AMK, AMC, AZM, CIP, ETP, EMB, IHN, LZD, MEM, PZA, RFP, TGC for 7 years | Persistent | IFN-γ | ··· | ··· | ··· | Remission |
|  |  |  |  |  | Immunoglobulin | ··· | ··· | ··· |  |
|  |  |  |  |  | Plasmapheresis | ··· | ··· | ··· |  |
|  |  |  |  |  | RTX: 375 mg/m^2^/week, 8 doses | 73.7% decrease | ··· | 2 |  |
| P6 ^[11]^ | F, 50 | *M. avium* | CLR, EMB, MXFX for 1 year | Persistent | IFN-γ | ··· | ··· | ··· | Remission |
|  |  |  |  |  | RTX; 375 mg/m^2^/week, 3 doses | 65% decrease | ··· | ··· |  |
| P7 ^[11]^ | F, 60 | *M. intracellulare* | AMK, AZM, CLR, EMB, INH, LXFX, MXFX, PZA, RFP for 1 year | Persistent | IFN-γ | ··· | ··· | ··· | Remission |
|  |  |  |  |  | RTX: 375 mg/m^2^/week, 7 doses | Decrease 58% | ··· | ··· |  |
| P8 ^[12]^ | M, 78 | EBV,  *M. chelonae-abscessus* | AZM, IPM, TOB, MXFX, CFZ for 3 years | Relapse | RTX: 375 mg/m^2^,  MP: 100 mg/7 days for 8 months | ··· | CD20 cell depletion (<1%), VZV and CMV | 8 | Remission |
| P9 ^[13]^ | M, 59 | *M. abscessus* | CLR, DOX, CTB for 2 months | Deteriorate | Dexamethasone for 1 month RTX for 4 courses | ··· | ··· | 12 | Remission |
| P10 ^[14]^ | M, 72 | *M. abscessus*,  *T. marneffei* | IHN, RFP, PZA, EMB, AMK, IPM, CLR, CIP for 9 months, LAMB followed by oral ICZ for 6 months | Persistent | MP: 1000 mg,  RTX: 375 mg/m^2^/week, 16 doses | 1:10 000 decreased to 1:5000 | ··· | 12 | Remission |
| P11 ^[15]^ | F, 67 | MAC | RFP, CLR, EMB, STFX, AMK, LZD, AZM, LVFX for 7 months | Deteriorated | RTX: 375 mg/m^2^/w, 4 doses | 173 E.U decreased to 51.7 E.U. | Leukocyto-penia | 4 | Remission |
| P12 ^[16^**^]^** | F, 30 | MAC | RFP, EMB, CLR | ··· | RTX: 375 mg/m^2^ and MP 100 mg for 6 mo | ··· | ··· | ··· | Remission |
| P13 ^[17]^ | F, 48 | MAC | ··· | Persistent | RTX: 500 mg/dose/2weeks, 4 cycles, cumulative dose: 339 mg/m^2^ | ··· | CD20 cell depletion | 66 | Remission |
| P14 ^[17]^ | F, 44 | *M. szulgai*,  *M. angelicum* | ··· | Persistent | RTX: 500 mg/dose/2weeks, 4 cycles, cumulative dose: 359 mg/m^2^ | ··· | CD20 cell depletion | 8 | Remission |
| P15 ^[17]^ | F, 79 | *M. avium*, MAC | ··· | Persistent | RTX: 500 mg/dose/2weeks, 2 cycles, cumulative dose: 326 mg/m^2^ | ··· | CD20 cell depletion | 12 | Remission |
| P16 ^[17]^ | M, 51 | MAC | ··· | Persistent | RTX: 500 mg/dose/2weeks, 2 cycles, cumulative dose: 319 mg/m^2^ | ··· | CD20 cell depletion | 19 | Remission |
| P17 ^[17]^ | M, 62 | *Salmonella* | ··· | Persistent | RTX: 500 mg/dose/2weeks, 2 cycles, cumulative dose: 286 mg/m^2^ | ··· | CD20 cell depletion | 29 | Remission |
| P18 ^[18]^ | F, 44 | CMV, *Salmonella*, *M. intracellulare*, *M. abscessus*,  *M. goodii* | AZM, RFP, EMB, CXM, GCV | Persistent | RTX: 1000 mg/dL, d15 for 6 months | ··· | ··· | ··· | Remission |
| P19 ^[19]^ | F, 38 | MAC *M. intracellulare* | AZM, EMB, AMK, RFP, LZD | Persistent | RTX: 1000 mg on days 0, 14, 42 for 8 doses | Decrease | CD20 cell depletion | 31 | Persistent |
|  |  |  |  |  | Bortezomib: 1.3 mg/m^2^ on days 1, 4, 8, and 11 per cycle | Decrease | Injection site irritation and gastro-intestinal discomfort. | 25 | Remission |
| P20 ^[20]^ | F, 31 | *M. avium* | AZM, EMB, MXFX, TZD, CFZ | Persistent | RTX 1000 mg/month for 5 months  Bortezomib: 1.3 mg/m^2^ | Decrease | CD20 cell depletion and elevated AST and ALT levels | 8 | Remission |
|  |  |  |  |  | Daratumumab:16 mg/kg/week for 5 doses | Decrease | ··· | 5 |  |
| P21 ^[21]^ | F, 44 | MAC | AZM, EMB, RFB for 4 months | Deteriorated | RTX: 375 mg/m^2^/mo for 2 yr | Decreased | ··· | 30 | Remission |
| P22 ^[21^**^]^** | F, 53 | MAC | AZM, RFP, AMK, MXFX for 1 mo | Deteriorated | RTX: 375 mg/m^2^/w for 22 mo | ··· | ··· | ··· | Remission |
| P23 ^[21]^ | M, 53 | *Salmonella*, MAC,  *M. abscessus* | AZM, RFB, EMB, MXFX, AMK, LZD, FOX, CFZ for 18 mo | Relapsed | RTX: 375 mg/m^2^ (at months 0, 1, 6, and 12) for 2 years | 50% decrease | VZV | 30 | Remission |
| P24 ^[22]^ | F, 78 | M. tuberculosis, MAI | AMK, AZM, CIP，ETP，INH, RFP, MEM, PZA, TGC | Persistent | RTX for 5 years | Decreased | ··· | ··· | Remission |
| P25 ^[23]^ | M, 77 | MAC | CLR, RFP, EMB, STFX for 4 yr | Persistent | R-CHOP: 6 cycles | Decreased during the R-CHOP therapy | ··· | 36 | Remission |
| P26 ^[24]^ | F, 44y | MAC,  influenza A virus | RFP, SM, EMB, CLR, MFLX for 2 mo | Deteriorated | CTX: 0.4 g/kg/dose for 5 doses | No decrease | ··· | ··· | Remission |
| P27 ^[25]^ | F, 38 | MAI | RFP, EMB, AZM for 6 mo | Persistent | Plasmapheresis, CTX, prednisolone for 1.5 years | Decreased | ··· | 18 | Remission |
| P28 ^[26]^ | M, 52 | NTM, VZV,  *T. marneffei,  M. tuberculosis* | CLR, OFLX, IPM, FOX for 19 months | Persistent | CTX: 400 mg/cycle for 5 cycles  MP: 1,000 mg followed by oral prednisolone 30 mg/day | 1:100,000 decreased to 1:50,000 | ··· | ··· | Death |
| P29 ^[26]^ | M, 54 | *M. abscessus* | CLR, CIP, IMP for 15 months | Persistent | CTX 400 mg/cycle for 14 cycles  MP: 1,000 mg followed by oral prednisolone 30 mg/d | 1:200,000 decreased to 1:5,000 | ··· | 57 | Remission |
| P30 ^[26]^ | M, 57 | *M. abscessus*,  *M. tuberculosis* | CIP, IPM, AZM for 10 months | Persistent | CTX 400 mg/cycle for 16 cycles  MP: 1,000 mg followed by oral prednisolone 30 mg/d | 1:200,000 decreased to 1:1,000 | ··· | 44 | Remission |
| P31 ^[26]^ | M, 41 | M. abscessus, *Burkholderia pseudomallei* (melioidosis) | CLR, OFLX, IMP for 19 months | Persistent | CTX 400 mg/cycle for 17 cycles  MP: 1,000 mg followed by oral prednisolone 30 mg/d | 1:200,000 decreased to 1:1,000 | ··· | 60 | Remission |
| P32 ^[26]^ | F, 54 | *M. abscessus* | CLR, CIP, IPM for 12 months | Persistent | CTX 400 mg/cycle for 17 cycles  MP: 1,000 mg followed by oral prednisolone 30 mg/d | 1:100,000 decreased to 1:1,000 | ··· | 28 | Remission |
| P33 ^[26^**^]^** | M, 65 | *M. abscessus*, *Salmonella* | CLR, CIP, IMP for 48 months | Persistent | CTX 400 mg/cycle for 17 cycles  MP: 1,000 mg followed by oral prednisolone 30 mg/d | 1:100,000 decreased to 1:5,000 | ··· | 44 | Remission |
| P34 ^[26]^ | M, 34 | *M. abscessus*, *Cryptococcus*,  *M. tuberculosis* | CLR, CIP, IMP, LZD for 15 months | Persistent | CTX 400 mg/cycle for 25 cycles  MP: 1,000 mg followed by oral prednisolone 30 mg/d | 1:200,000 decreased to 1:50,000 | ··· | 45 | Relapse |
| P35 ^[26]^ | F, 20 | *M. abscessus*, *Cryptococcus*, VZV | AZM, OFLX, DOX, LZD, IMP for 26 months | Persistent | CTX 400 mg/cycle for 20 cycles  MP: 1,000 mg followed by oral prednisolone 30 mg/d | 1:400,000 decreased to 1:50,000 | ··· | 41 | Remission |
| P36 ^[17]^ | F, 35 | MAC,  *M. fortuitum* | ··· | Persistent | CTX: 5–15 mg/kg/dose, 13 cycles, cumulative dose: 11250 mg | ··· | ··· | 64 | Remission |
| P37 ^[17]^ | M, 59 | *M. kansasii*, *Salmonella*, HSV, *Cryptococcus* | ··· | Persistent | CTX: 5–15 mg/kg/dose, 10 cycles, cumulative dose: 7500 mg | ··· | ··· | 58 | Remission |
| P38 ^[17]^ | F, 56 | NTM | ··· | Persistent | CTX: 5–15 mg/kg/dose, 8 cycles, cumulative dose: 4100 mg | ··· | ··· | 30 | Remission |
| P39 ^[17^**^]^** | M, 59 | *M. abscessus*，VZV | ··· | Persistent | CTX: 5–15 mg/kg/dose, 6 cycles, cumulative dose: 4600 mg | ··· | ··· | 36 | Remission |
| P40 ^[17]^ | M, 40 | *M. abscessus* | ··· | Persistent | CTX: 5–15 mg/kg/dose, 10 cycles, cumulative dose: 7400 mg | ··· | ··· | 53 | Remission |
| P41 ^[17]^ | F, 47 | *M. manteneii*，Cryptococcus | ··· | Persistent | CTX: 5–15 mg/kg/dose, 6 cycles, cumulative dose: 3350 mg | ··· | ··· | 30 | Remission |
| P42 ^[17]^ | F, 45 | *M. parascofulaceum*,  *M. abscessus* | ··· | Persistent | CTX: 5–15 mg/kg/dose, 5 cycles, cumulative dose: 3650 mg | ··· | ··· | 28 | Remission |
| P43 ^[17]^ | F, 66 | *Salmonella*, VZV | ··· | Persistent | CTX: 500 mg/dose/2weeks, 6 cycles, cumulative dose: 345 mg mg/m^2^ | ··· | CD20 cell depletion | 8 | Remission |
| P44 ^[27]^ | M, 55 | *M. intracellulare* | CLR, EMB, RFP | Persistent | Prednisolone (10 mg/day), adalimumab 40 mg/2 weeks | ··· | ··· | ··· | Relapse |
| P45–P52 ^[28]^ | ··· | ··· | ··· | Persistent | CTX, 8 cases | Decreased | ··· | ··· | 1 death, 7 remission |
| P53–P62 ^[28]^ | ··· | ··· | ··· | Persistent | RTX 10 cases | Decreased | ··· | ··· | 10 remission |
| P63 | M, 63 | TM, *M.chelonae* | MXFX, CLR,EMB, for 7 months,  AMB+ICZ for 3 months followed by oral ICZ for 6 months | Relapse | CTX: 5–15 mg/kg/dose,  6 cycles, cumulative dose: 5.8g | Decreased | CD20 cell depletion | 24 | Remission |
| P64 | M, 62 | TM | VCZ for 8 months | Relapse | CTX: 5–15 mg/kg/dose,  6 cycles, cumulative dose: 5.5g | Decreased | CD20 cell depletion | 24 | Remission |
| P65 | F, 43 | TM, *M.abscessus*  *Salmonella* | VCZ for 2 months  MXFX, CLR,EMB, for 4 months | Relapse | CTX: 5–15 mg/kg/dose,  6 cycles, cumulative dose: 5.5g | Decreased | CD20 cell depletion | 24 | Relapse |
| P66 | F, 33 | TM, *M.abscessus* | MXFX, CLR,EMB, for 4 months,  ICZ for 2 months | Relapse | CTX: 5–15 mg/kg/dose,  6 cycles, cumulative dose: 4.8g | Decreased | CD20 cell depletion | 24 | Remission |
| P67 | M,64 | TM, *Salmonella* | VCZ for 4 months  piperacillin/tazobactam+LXFX | Relapse | CTX: 5–15 mg/kg/dose,  6 cycles, cumulative dose: 5.6g | Decreased | CD20 cell depletion | 24 | Remission |

AIGA, anti-interferon-γ autoantibodies; ALT, alanine aminotransferase; AMC, amoxicillin/clavulanate; AMK, amikacin; AST, aspartate aminotransferase; AZM, azithromycin; CIP, ciprofloxacin; CFZ, clofazimine; CLR, clarithromycin; CMV, cytomegalovirus; CTB, ceftibuten; CTX, cyclophosphamide; CXT, cefoxitin; DOX, doxycycline; EBV, Epstein-Barr virus; EMB, ethambutol; ETP, ertapenem; FOX, cefoxitin; GCV, ganciclovir; HSV, human herpesvirus; HZV, ICZ, itraconazole; IMP, imipenem; INH, isoniazid; LAMB, amphotericin B liposome; LXFX, levofloxacin; LZD, linezolid; MAC, *Mycobacterium avium* complex MAI: *Mycobacterium avium-intercellulare*; MEM, meropenem; MP, methylprednisolone, MXFX, moxifloxacin; NTM, nontuberculous mycobacteria; OFLX, ofloxacin; PZA, pyrazinamide; R-CHOP, rituximab, cyclophosphamide, hydroxydaunorubicin hydrochloride (doxorubicin hydrochloride), vincristine (Oncovin) and prednisone; RFP, rifampin; RTX, rituximab; SM, streptomycin; SMZ, sulfamethoxazole; STFX, sitafloxacin; *T. marneffei*, *Talaromyces marneffei*; TGC, tigecycline; TOB, tobramycin; TZD, tedizolid; VZV, varicella zoster virus. P63 to P67 were the participants in the retrospective study.
